# Uncovering the effects and molecular mechanism of *Astragalus membranaceus* (Fisch.) Bunge and its bioactive ingredients formononetin and calycosin against colon cancer: An integrated approach based on network pharmacology analysis coupled with experimental validation and molecular docking

**DOI:** 10.3389/fphar.2023.1111912

**Published:** 2023-01-23

**Authors:** Yu Hu, Wenjuan Zhai, Duanling Tan, Haipeng Chen, Guiyu Zhang, Xuanjing Tan, Yuting Zheng, Wenhui Gao, Yijie Wei, Jinjun Wu, Xin Yang

**Affiliations:** ^1^ Guangdong Provincial Key Laboratory of Translational Cancer Research of Chinese Medicines, Joint International Research Laboratory of Translational Cancer Research of Chinese Medicines, School of Pharmaceutical Sciences, International Institute for Translational Chinese Medicine, Guangzhou University of Chinese Medicine, Guangzhou, Guangdong, China; ^2^ Key Laboratory of Molecular Target and Clinical Pharmacology and the State, NMPA Key Laboratory of Respiratory Disease, School of Pharmaceutical Sciences and The Fifth Affiliated Hospital, Guangzhou Medical University, Guangzhou, China; ^3^ Affiliated Cancer Hospital and Institute of Guangzhou Medical University, Guangzhou, Guangdong, China

**Keywords:** colon cancer, *Astragalus membranaceus* (Fisch.) Bunge, formononetin, calycosin, network pharmacology, ERK1/2

## Abstract

Colon cancer is a highly malignant cancer with poor prognosis. *Astragalus membranaceus* (Fisch.) Bunge (Huang Qi in Chinese, HQ), a well-known Chinese herbal medicine and a popular food additive, possesses various biological functions and has been frequently used for clinical treatment of colon cancer. However, the underlying mechanism is not fully understood. Isoflavonoids, including formononetin (FMNT) and calycosin (CS), are the main bioactive ingredients isolated from HQ. Thus, this study aimed to explore the inhibitory effects and mechanism of HQ, FMNT and CS against colon cancer by using network pharmacology coupled with experimental validation and molecular docking. The network pharmacology analysis revealed that FMNT and CS exerted their anticarcinogenic actions against colon cancer by regulating multiple signaling molecules and pathways, including MAPK and PI3K-Akt signaling pathways. The experimental validation data showed that HQ, FMNT and CS significantly suppressed the viability and proliferation, and promoted the apoptosis in colon cancer Caco2 and HT-29 cells. HQ, FMNT and CS also markedly inhibited the migration of Caco2 and HT-29 cells, accompanied by a marked increase in E-cadherin expression, and a notable decrease in N-cadherin and Vimentin expression. In addition, HQ, FMNT and CS strikingly decreased the expression of ERK1/2 phosphorylation (p-ERK1/2) without marked change in total ERK1/2 expression. They also slightly downregulated the p-Akt expression without significant alteration in total Akt expression. Pearson correlation analysis showed a significant positive correlation between the inactivation of ERK1/2 signaling pathway and the HQ, FMNT and CS-induced suppression of colon cancer. The molecular docking results indicated that FMNT and CS had a strong binding affinity for the key molecules of ERK1/2 signaling pathway. Conclusively, HQ, FMNT and CS exerted good therapeutic effects against colon cancer by mainly inhibiting the ERK1/2 signaling pathway, suggesting that HQ, FMNT and CS could be useful supplements that may enhance chemotherapeutic outcomes and benefit colon cancer patients.

## 1 Introduction

Colon cancer is among the most prevalent cancers, which has a complex aetiology consisting of environmental component and genetic component (Fagunwa et al., 2017; Kong et al., 2020). Evidence exists that colon cancer arises from two types of precursor polyps *via* two distinct pathways: conventional adenomas by the conventional adenoma-to-carcinoma sequence and serrated adenomas according to the serrated adenoma-to-carcinoma theory (Cappell, 2008). Currently, colon cancer is the third most common diagnosis and second deadliest malignancy for both sexes combined. It is estimated that colon cancer will cause 1.1 million deaths by 2030 (Kong et al., 2020). Current clinical treatment of colon cancer mainly includes operation, radiotherapy and chemotherapy. Despite advances in the development of these therapies, the prognosis in colon cancer patients remains poor and the recurrence rate is still high (Kong et al., 2020). Moreover, long-term use of these therapies easily produces severe toxicities side effects, including hepatotoxicity, vomiting, diarrhea and immunosuppression (Kong et al., 2020). Thus, alternative therapies with pronounced effectiveness but low toxicity are necessary.

Nowadays, the interest in Chinese herbal medicines (CHMs) has increased worldwide due to their health-beneficial properties. The obvious advantages of CHMs in the prevention and treatment of colon cancer has been widely recognized and confirmed (Ye et al., 2015; Yan et al., 2017; Kong et al., 2020). *Astragalus membranaceus* (Fisch.) Bunge (Huang Qi in Chinese, HQ), one of the most well-known CHMs, is frequently used as a major component in over 200 Chinese medicine formulas to treat a wide variety of diseases and body disorders, including chronic fatigue, weakness, wounds, anemia, fever, uterine bleeding, allergies and cancer (Guo et al., 2019; Lou et al., 2019). Moreover, as a health-beneficial additive in some food, HQ is popularly consumed to strengthen the immune system and reinforce vital energy (Guo et al., 2019). Pharmacological evidence revealed that HQ manifests various biological functions, such as anti-inflammation, anti-oxidant, antiviral, immunomodulation, and anti-carcinogenic activities without any appreciable toxicity (Guo et al., 2019; Lou et al., 2019; An et al., 2020). Previous studies demonstrated that HQ has a utility in clinical applications for the colon cancer treatment. For example, HQ could not only inhibit the proliferation, cause cell cycle arrest and promote apoptosis of colon cancer cells, but also suppress the migration of colon cancer cells (Tseng et al., 2016; Tan et al., 2019). HQ could effectively inhibit the tumors of human colon cancer cells grafted into nude mice without obvious side effect (Tseng et al., 2016), which demonstrated that HQ might be a potential chemopreventive drug for colon cancer therapy. What is more, HQ exerted potent anti-tumor activity through simultaneous modulation of mammalian target of rapamycin (mTOR) and cyclooxygenase-2 (COX-2) signaling, which together decreased vascular endothelial growth factor (VEGF) expression to effectively suppress angiogenesis in tumor xenograft and human colon cancer cells (Auyeung et al., 2016). Although increasing evidence supports the potent inhibitory activity of HQ against colon cancer, the molecular mechanism underlying the action has yet to be fully elucidated. Moreover, colon cancer is a systemic and complicated disease with involvement of multiple signaling molecules. It is essential to further investigate the underlying mechanisms of HQ in the suppression of colon cancer.

Numerous ingredients have been isolated and identified from HQ (Guo et al., 2019). Formononetin (FMNT) and calycosin (CS), two main bioactive isoflavone ingredients isolated from HQ, contribute to the therapeutic efficacy and pharmacological activities of HQ (Guo et al., 2019). It was reported that FMNT could suppress the growth and induce apoptosis of colon cancer cells, which were associated with caspase activation and decrease in protein levels of Bcl-2 and Bcl-x(L). FMNT also inhibited angiogenesis and invasion of colon cancer cells, thereby supporting its potential application in advanced and metastatic colon therapies (Auyeung et al., 2012). CS, as an important marker for monitoring the HQ quality, is pharmacologically beneficial due to its antioxidative, cytoprotection, hypolipemic, neuroprotection, and hypoglycemic effects (Gong et al., 2021; Li et al., 2022). Moreover, CS has potent anti-carcinogenic activities, including action against colon cancer. It was reported that CS notably inhibited proliferation and induced apoptosis of colon cancer cells through regulating the ERβ/MiR-95, IGF-1R and PI3K-Akt signaling pathways (Zhao et al., 2016). CS also promoted apoptosis of human colon cancer cells through modulation of ERβ/miR-17 signaling pathway (Chen et al., 2015b). These studies indicate that FMNT and CS may be used as candidate agents for clinical treatment of colon cancer in the future. However, the network molecular mechanisms of FMNT and CS against colon cancer has not been well defined.

**FIGURE 1:**
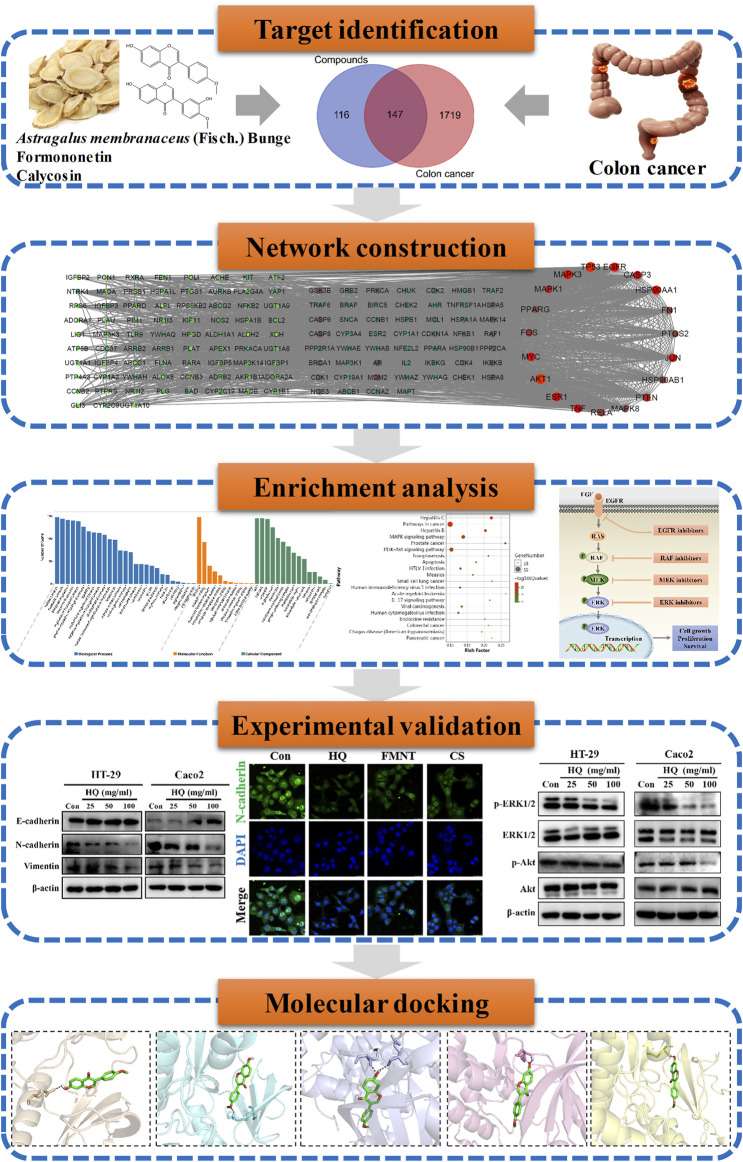
Technological road-map. The whole technical roadmap was divided into five steps, including target identification, network construction, enrichment analysis, experimental validation and a molecular docking study.

Network pharmacology is an emerging methodology that attempts to eliminate drug actions and reveal the interactions between drugs and targets (Hopkins, 2007). As a powerful tool, network pharmacology can combine network biology and polypharmacology approaches to provide evidence for the molecular targets and the potential mechanisms of action of CHMs (Gan et al., 2019; An et al., 2020). Thus, this current study integrates network pharmacology with experimental validation and molecular docking to clarify the effects and potential mechanism of HQ, FMNT and CS against colon cancer (Figure 1). The objectives of the current study were: 1) to determine the content of FMNT and CS in the water extract of HQ for quality control; 2) to screen the potential targets of FMNT and CS against colon cancer; 3) to explore the potential mechanism of FMNT and CS against colon cancer by using network pharmacology; 4) to evaluate the inhibitory effects of HQ water extract, FMNT and CS against colon cancer HT-29 and Caco-2 cells, and to elucidate the potential mechanism by using experimental validation; 5) to investigate the binding ability between FMNT and CS and key targets by using molecular docking. Our results may expand our current knowledge about the mechanism of HQ as a therapeutic candidate for colon cancer therapy.

## 2 Materials and methods

### 2.1 Preparation of HQ water extract and UHPLC-MS/MS analysis

**FIGURE 2:**
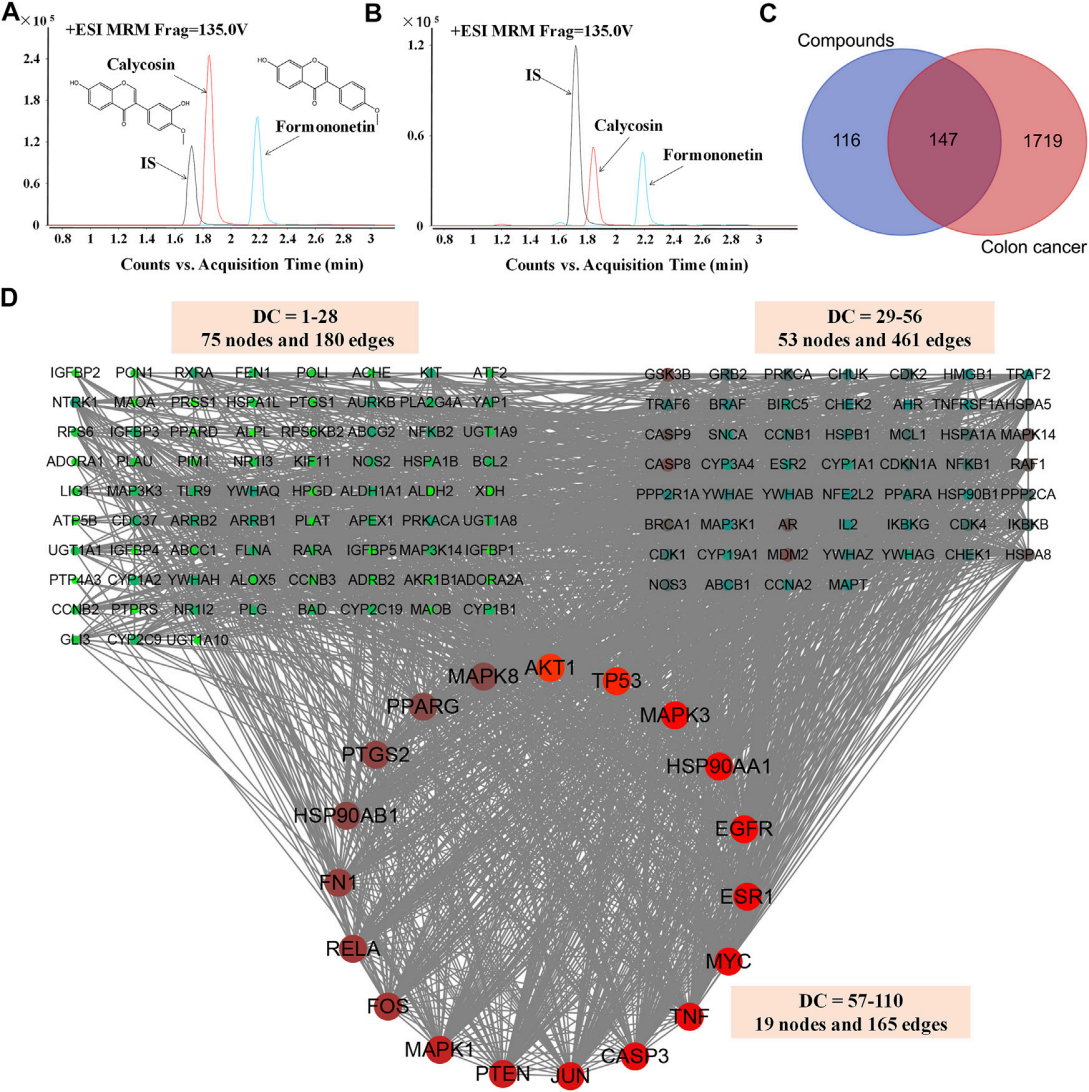
Screening and analysis of the common targets of FMNT and CS for the treatment of colon cancer. The content of FMNT and CS were quantitated in HQ water extract using erlotinib as an internal standard (IS) **(A)** A chromatogram of FMNT and CS standard solution **(B)** A chromatogram of FMNT and CS in HQ water extract **(C)** Venn diagram of compounds (FMNT and CS)-colon cancer intersection targets. The 1866 targets of colon cancer were mapped to the 263 targets of FMNT and CS to screen out the 147 common targets **(D)** The PPI network of the 147 nodes. The red nodes represent the big hub nodes, the green nodes represent the other nodes. The node size is proportional to its degree.

One hundred-gram pieces of HQ were weighed. Preparation of HQ water extract (1 g/mL) were performed as previously described (Shi et al., 2015; Lou et al., 2019). Chemical profiling and standardization of HQ water extract using FMNT and CS was performed *via* UHPLC-MS/MS analysis by using an Agilent 1290 infinity LC system (Supplementary materials and methods). The content of FMNT and CS were quantitated in HQ water extract at levels of 0.3338 mg/g and 0.1998 mg/g, respectively (Figures 2A, B).

### 2.2 Prediction of the targets for FMNT and CS

Several databases, including TCMSP (http://tcmspw.com/) (Tan et al., 2022), SuperPred (http://prediction.charite.de/) (An et al., 2020), SwissTargetPrediction (http://www.swisstargetprediction.ch/) (Tan et al., 2022), PubChem (https://pubchem.ncbi.nlm.nih.gov/) (An et al., 2020) and STITCH (http://stitch.embl.de/) (An et al., 2020) databases, which limit species for “*Homo sapiens*”, were used to collect the potential targets of FMNT and CS. Finally, the UniProt database (https://www.uniprot.org/) was used to identify all the targets (An et al., 2020).

### 2.3 Screening of potential targets for colon cancer

Keywords of “colon cancer”, “colon adenocarcinoma” and “colorectal cancer” were used to identify targets related to colon cancer from GeneCards (https://www.genecards.org/) (Tan et al., 2022), OMIM (https://www.omim.org/) (Amberger et al., 2015), TTD (http://db.idrblab.net/ttd/) (Tan et al., 2022), DisGeNET (https://www.disgenet.org/) (Tan et al., 2022) and CTD (http://ctdbase.org) (Tan et al., 2022) databases. Finally, the UniProt database (https://www.uniprot.org/) was used to identify all the targets (An et al., 2020).

### 2.4 Network construction and analysis

Venn diagram (http://bioinformatics.psb.ugent.be/webtools/Venn/) was used to collect the common targets of FMNT and CS and colon cancer. The detectable targets in the protein-protein interaction (PPI) of FMNT and CS against colon cancer were constructed by the STRING database (https://string-db.org/) and were further visualized by using Cytoscape software (An et al., 2020; Xu et al., 2022). Crucial biotargets of FMNT and CS against colon cancer were obtained according to the degree values in the Cytoscape settings.

### 2.5 Gene ontology and pathway enrichment analysis

The Omicshare Online tools (https://www.omicshare.com/tools), a free online platform for data analysis, was used to understand the role of the potential targets in gene functions and signaling pathways, including the Gene Ontology (GO) and Kyoto Encyclopedia of Genes and Genomes (KEGG) pathway enrichment analysis (An et al., 2020). GO (https://geneontology.org/) is one of the main resources of biological information, which provides a specific definition of protein functions. GO is subdivided into three non-overlapping ontologies: biological process (BP), cellular component (CC), and molecular function (MF) terms (Ashburner et al., 2000). Those terms with a *p* < 0.05 were chosen for further analysis and the top 20 of GO terms enrichment were displayed. KEGG (https://www.kegg.jp/) is a database resource for the biological interpretation by genome sequencing and other high-throughput experimental technologies (Chen et al., 2015a; An et al., 2020). Those terms with an *p* < 0.05 were chosen for further analysis and the top 25 enriched KEGG pathways were mapped.

### 2.6 Chemicals and reagents

*Astragalus membranaceus* (Fisch.) Bunge was purchased from Daxiang Chinese Pharmaceutical Co., Ltd. (Guangdong, China). FMNT and CS (purity ≥98%) were purchased from Chengdu Must Biotechnology Co., Ltd. (Chengdu, China). MTT were bought from Solarbio Science and Technology Co., Ltd. (Beijing, China). BeyoClick™ EdU cell proliferation kitwas bought from Beyotime Biotechnology (Haimen, China). Annexin V-fluorescein isothiocyanate (FITC)/propidium iodide (PI) apoptosis detection kit was purchased from Absin Bioscience Inc. (Shanghai, China). DMSO and HEPES were purchased from GBCBIO Technologies (Guangzhou, China). p-Akt (Ser473); Akt, p-ERK1/2 (Thr202/Tyr204), ERK1/2, and β-actin antibodies were purchased from Cell Signaling Technology (Danvers, MA, United States). N-cadherin, E-cadherin and Vimentin antibodies were purchased from Affinity Biosciences (OH, United States). All other chemicals not mentioned here were of analytical reagent grade or better.

### 2.7 Cell culture

HT29 and Caco2 human colon carcinoma cells were purchased from American Type Culture Collection (ATCC, Manassas, VA, United States) and then routinely cultured in DMEM medium (Gibco, United States) containing 10% (v/v) fetal bovine serum (FBS) (Gibco, United States) and 100 U/mL streptomycin-penicillin solution (Gibco, United States) in a humidified atmosphere containing 5% CO_2_ at 37°C.

### 2.8 MTT assay

Cell viability was measured by MTT assay. HT29 and Caco2 cells were seeded in 96-well plates and cultured with the vehicle, HQ (3.125–100 mg/mL), FMNT (3.125–200 μM) or CS (3.125–200 μM) for 48 h. 5-Fluorouracil (5-FU) (25 μM) was used as a positive control. Then, the medium was removed and each well was added 200 μL MTT solution (0.5 mg/mL) and incubated at 37°C for additional 4 h. After that, supernatant was discarded and 150 μl DMSO was added to each well to dissolve formazan crystals. Optical density (OD) values were measured at 490 nm by a microplate reader (PerkinElmer, Waltham, MA, United States). Finally, cell inhibitory rates and 50% inhibitory concentration (IC_50_) values were calculated for each group.

### 2.9 5-Ethynyl-2′-Deoxyuridine (EdU) assay

Cell proliferation ability was measured by EdU assay following the manufacturer’s instructions. HT29 and Caco2 cells were seeded in 96-well plates and cultured with the vehicle, HQ (25, 50, and 100 mg/mL), FMNT (25, 50, and 100 μM) or CS (25, 50, and 100 μM) for 48 h. 5-FU (25 μM) was used as a positive control. Then, cells were incubated with EdU labeling medium (50 mM) at 37°C for 2 h. Other details of the procedure are described in literature (Wu et al., 2020). Finally, the images were captured using a fluorescence microscope (Leica, Germany) and analyzed by ImageJ software.

### 2.10 Wound healing assay

HT29 and Caco2 cells were seeded in 6-well plates. Scratch lines were drawn on the monolith of the fused cells using 10 μL pipette tips. Then, the cells were cultured with the vehicle, HQ (25, 50, and 100 mg/mL), FMNT (25, 50, and 100 μM) or CS (25, 50, and 100 μM) for 12, 24 or 48 h, respectively. Pictures of the scratch lines were captured using a microscope (Leica, Germany) at 0, 12, 24 and 48 h, respectively. The cell migration ratio of linear wounds was calculated by using ImageJ software.

### 2.11 Cell apoptosis assay

HT29 and Caco2 cells were stained with Annexin V-FITC/PI and evaluated for apoptosis by flow cytometry according to the manufacturer’s instructions. Briefly, HT29 and Caco2 cells were seeded in 6-well plates and exposed to the vehicle, HQ (25, 50, and 100 mg/mL), FMNT (25, 50, and 100 μM) or CS (25, 50, and 100 μM) for 48 h. Then, cells were collected and stained with Annexin V-FITC and PI for 20 min at room temperature in dark. Finally, cell apoptosis was analyzed by the flow cytometry (BD Biosciences, San Diego, CA, United States).

### 2.12 Western blot assay

HT29 and Caco2 cells were seeded in 6-well plates and exposed to the vehicle, HQ (25, 50, and 100 mg/mL), FMNT (25, 50, and 100 μM) or CS (25, 50, and 100 μM) for 48 h. After that, the cells were washed with cold PBS and harvested. Total proteins were extracted from cells using the RIPA lysis reagent containing a protease inhibitor and then quantified using a BCA estimation kit. Western blotting was performed as previously described (An et al., 2020) using primary antibodies against p-Akt, Akt, p-ERK1/2, ERK1/2, N-cadherin, E-cadherin, Vimentin or β-actin (1:1000). Protein bands were detected by a gel imaging analysis system and were analyzed using ImageJ software.

### 2.13 Immunofluorescence

HT29 and Caco2 cells were seeded on confocal dishes and exposed to the vehicle, HQ (100 mg/mL), FMNT (100 μM) or CS (100 μM) for 48 h. At the end of the incubation, the cells were fixed in paraformaldehyde, permeabilized with TritonX-100 and blocked with bovine serum albumin. Then, the cells were incubated with a N-cadherin, an E-cadherin, or a Vimentin (1:200) antibody at 4°C overnight and then stained with a secondary fluorescent antibody (1:200; Alexa Fluor 488). Finally, the cells were incubated with DAPI for 20 min. Fluorescence signals were detected using a Leica TCS SP8 confocal fluorescence microscope (Leica, Germany). The relative fluorescence of N-cadherin, E-cadherin and Vimentin were analyzed by ImageJ software.

### 2.14 Molecular docking

Molecular docking was carried out by AutoDock Vina software to measure the interactions between the two compounds and the key upstream molecules of ERK1/2 signaling pathway, including EGFR, BRAF, MEK1/2 and ERK1/2. The crystal structures of EGFR, BRAF, MEK1/2 and ERK1/2 were retrieved from the Protein Data Bank (Table 1). The mol2 file format structures of the FMNT and CS were obtained from the PubChem database. Details of the procedure are described in literature (Ruan et al., 2020). After docking, conformations with the lowest docking energy were selected for the binding conformations between the ligands (FMNT and CS) and the target proteins. Finally, the analysis and visualization of the ligand-protein complexes were performed by PyMOL software.

**TABLE 1:** Molecular docking of FMNT and CS and corresponding targets.

Targets	Protein data bank ID	Compounds	Binding energy (kcal/mol)
**EGFR**	1M17	formononetin	−7.9
**EGFR**	1M17	calycosin	−8.3
**BRAF**	4EHG	formononetin	−9.2
**BRAF**	4EHG	calycosin	−9.5
**MEK1**	7B9L	formononetin	−8.5
**MEK1**	7B9L	calycosin	−9.0
**MEK2**	1S9I	formononetin	−7.8
**MEK2**	1S9I	calycosin	−8.3
**ERK1**	4QTB	formononetin	−9.3
**ERK1**	4QTB	calycosin	−9.6
**ERK2**	4ZZN	formononetin	−7.3
**ERK2**	4ZZN	calycosin	−7.3

### 2.15 Statistical analysis

All assays were performed in triplicate. Results were expressed as mean with standard deviations (mean ± SD). One-way analysis of variance (ANOVA) was performed to compare the differences between different groups by SPSS 19.0. Correlation analyses were performed using Pearson product–moment correlation by SPSS 19.0. Statistical significance of differences was accepted at *p* < 0.05, *p* < 0.01 and *p* < 0.001.

## 3 Results

### 3.1 Potential targets for compounds and colon cancer

A total of 263 potential targets for FMNT and CS were identified after screening the databases of TCMSP, SuperPred, SwissTargetPrediction, STITCH and PubChem, (Figure 2C). Through the GeneCards, OMIM, TTD, CTD and DisGeNET databases, a total of 1866 colon cancer targets were obtained (Figure 2C).

### 3.2 Network construction

Then, compound targets and colon cancer-related targets were mapped using a Venn diagram, 147 common targets were obtained as the potential targets of FMNT and CS against colon cancer (Figure 2C). The PPI network of the 147 targets were established by Cytoscape 3.7.2 software (An et al., 2020) (Figure 2D). There were 147 nodes and 2,382 edges in the PPI network with a medium degree centrality (DC) value of 28 (Figure 2D). Afterwards, hub nodes for FMNT and CS against colon cancer were established based on DC values. Finally, a total of 19 hub nodes were screened when the thresholds were set at DC ≥ 57. These 19 crucial targets with higher DC values were identified as the candidate targets of FMNT and CS against colon cancer, including AKT1, TP53, MAPK3, EGFR, HSP90AA1, ESR1, MYC, CASP3, TNF, JUN, PTEN, MAPK1, FOS, RELA, FN1, PTGS2, HSP90AB1, MAPK8 and PPARG (Figure 2D).

### 3.3 GO analysis and KEGG pathway enrichment analysis

**FIGURE 3:**
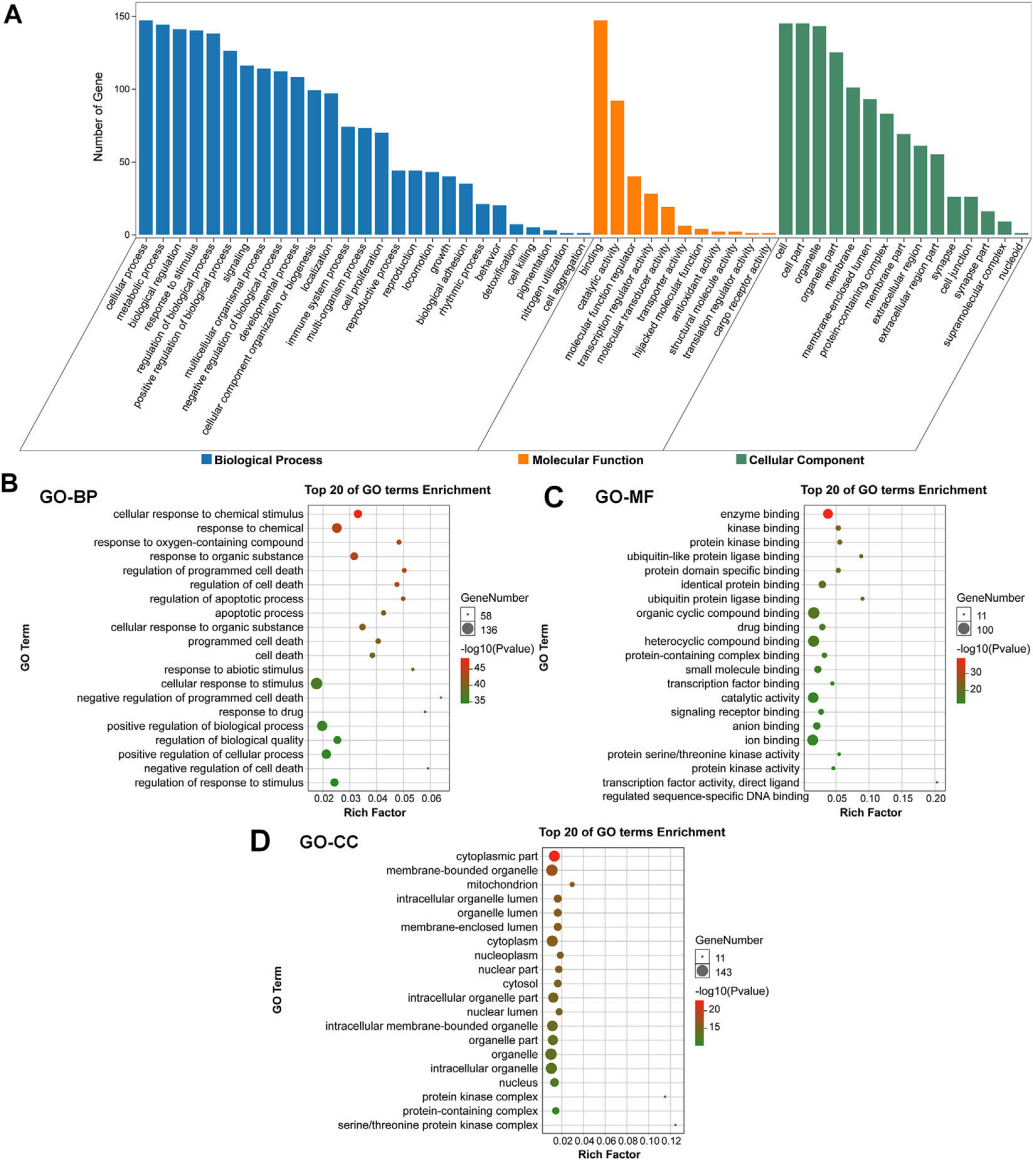
GO enrichment analysis of the key targets of FMNT and CS against colon cancer **(A)** The number of genes involved in the biological process (BP), molecular function (MF) and cellular component (CC) **(B)** GO-BP enrichment analysis of the 147 key targets **(C)** GO-MF enrichment analysis of the 147 key targets **(D)** GO-CC enrichment analysis of the 147 key targets. The top 20 enriched GO terms are displayed, and only terms with *p* < 0.05 were selected for analysis.

To evaluate the pharmacological effects of FMNT and CS against colon cancer. The potential 147 targets were further utilized for the GO analysis and KEGG pathway enrichment analysis. GO analysis showed that the majority of these 147 targets were enriched in cells with molecular function of protein binding. Specifically, these targets were abundantly enriched in cellular process, metabolic process, biological regulation and response to stimuli (Figure 3A). These results implicated that various cellular functions could be involved in the diverse and synergistic effects of FMNT and CS against colon cancer. The top 20 of GO terms enrichment were mapped (Figures 3B, C). The results showed that targets were closely related to multiple biological processes (BPs), including cellular response to chemical stimuli, response to chemical, response to oxygen-containing compounds, response to organic substances, regulation of programmed cell death, regulation of cell death, regulation of apoptotic process, and apoptotic process, etc (Figure 3B). In molecular functions (MFs), the significant terms were mainly enriched in enzyme binding, kinase binding, protein kinase binding, ubiquitin-like protein ligase binding, protein domain specific binding, identical protein binding, ubiquitin protein ligase binding, and organic cyclic compound binding, etc (Figure 3C). Moreover, cellular components (CCs) analysis showed that the significant terms mainly included cytoplasmic part, membrane-bounded organelle, mitochondrion, intracellular organelle lumen, organelle lumen, membrane-enclosed lumen, cytoplasm and nucleoplasm, etc (Figure 3D).

**FIGURE 4:**
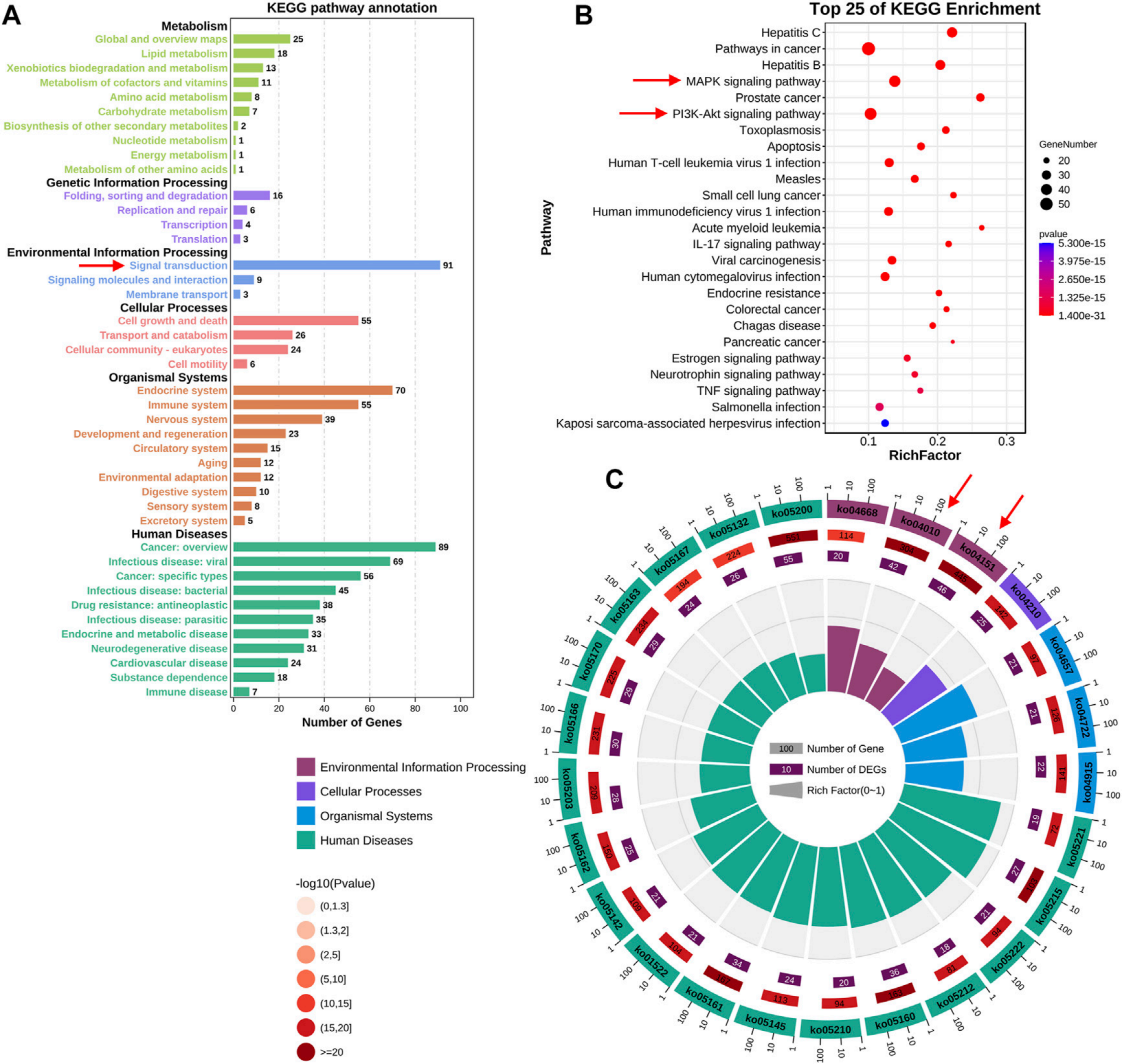
KEGG enrichment analysis of the key targets of FMNT and CS against colon cancer **(A)** The number of genes involved in the KEGG enrichment annotation **(B)** KEGG enrichment analysis of the 147 key targets. The top 25 enriched KEGG pathways are displayed, and only terms with *p* < 0.05 were selected for analysis **(C)** The number of genes and rich factors of the top 25 enriched KEGG pathways.

To further elucidate the potential mechanisms of FMNT and CS against colon cancer, KEGG pathway enrichment analysis of those 147 key targets was conducted. KEGG pathway annotation showed that 91 and 89 targets were involved in the signal transduction and cancer, respectively (Figure 4A). The top 20 KEGG pathway enrichments with a *p*-value <0.05 were displayed in Figure 4B. The significant pathways mainly included hepatitis C, pathways in cancer, hepatitis B, MAPK signaling pathway, prostate cancer, PI3K-Akt signaling pathway, toxoplasmosis and apoptosis, *etc.* It further showed that 42 and 46 targets were involved in the MAPK (ko04010) and PI3K-Akt (ko04151) signaling pathways, respectively (Figure 4C). The MAPK signaling pathway is essential in regulating many cellular processes including cell differentiation, cell stress response, cell division, metabolism, cell proliferation, motility and apoptosis. The role of the MAPK pathway in various diseases, including cancer, has been widely studied and well recognized (Haigis et al., 2008; Ahearn et al., 2011). As shown in Supplementary Figure 1, FMNT and CS could modulate multiple signaling molecules and pathways to suppress cancer proliferation, promote apoptosis, and inhibit angiogenesis. The key targets of the MAPK signaling pathway mainly include MAPK1, MAPK3, EGFR, AKT1, TP53, MYC, FOS, and MAPK8 (Supplementary Figure S1A). The PI3K-Akt signaling pathway is another crucial intracellular signal transduction pathway that closely regulate cell growth, migration, metabolism, proliferation and angiogenesis [28]. The PI3K-Akt is also a crucial signaling pathway contributing to initiation and development of various cancers (Noorolyai et al., 2019; Pompura and Dominguez-Villar, 2018). The key targets of the PI3K-Akt signaling pathway mainly include AKT1, HSP90AA1, HSP90AB1, TP53, PTEN, MYC, EGFR, and RELA (Supplementary Figure S1B). Hence, the potential mechanisms associated with the MAPK and PI3K-Akt signaling pathways were further verified using experimental validation coupled with molecular docking.

### 3.4 HQ, FMNT and CS significantly inhibited the viability of HT29 and Caco2 cells

**FIGURE 5:**
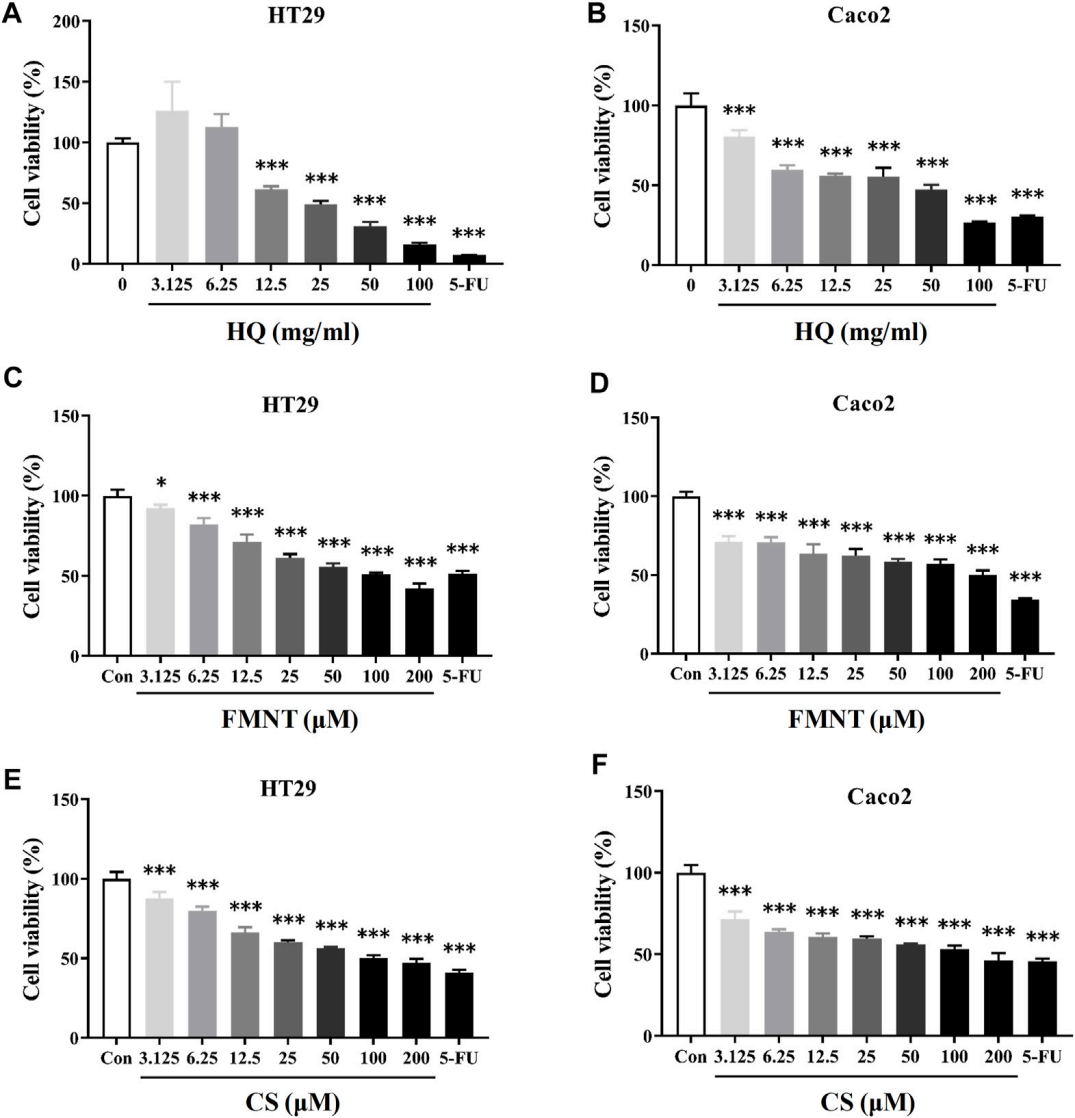
HQ, FMNT and CS significantly inhibited the viability of HT29 and Caco2 cells. The cytotoxicity of HQ (3.125–100 mg/mL, 48 h) **(A and B)**, FMNT (3.125–200 μM, 48 h) **(C and D)** and CS (3.125–200 μM, 48 h) **(E and F)** toward HT29 and Caco2 cells, respectively. 5-FU was used as a positive control for inhibition of cell viability. The data represent the mean ± SD (*n* = 3). ^*^ *p* < 0.05 and ^***^ *p* < 0.001 compared with the control group.

MTT assay was performed to detect the effects of HQ, FMNT and CS on the viability of HT29 and Caco2 cells. It was found that treatment with HQ at 3.125–100 mg/mL for 48 h significantly suppressed the viability of HT29 and Caco2 cells in a dose-dependent manner (Figures 5A, B, *p* < 0.001). The IC50 value of HQ in HT29 and Caco2 cells after treatment was 26.67 and 25.31 mg/mL, respectively. After treating cells with FMNT and CS at 3.125–200 μM for 48 h, cell viabilities were also significantly suppressed in a dose-dependent manner (Figures 5C–F, *p* < 0.05 or *p* < 0.001**)**. 5-FU, which was used a positive control, also produced significant inhibition toward the viability of HT29 and Caco2 cells as expected (*p* < 0.001). Accordingly, three concentrations of 25, 50, and 100 mg/mL for HQ, and three concentrations of 25, 50, and 100 μM for FMNT or CS, and a duration of 48 h treatment were selected to conduct the following experiments.

### 3.5 HQ, FMNT and CS significantly inhibited the proliferation of HT29 and Caco2 cells

**FIGURE 6:**
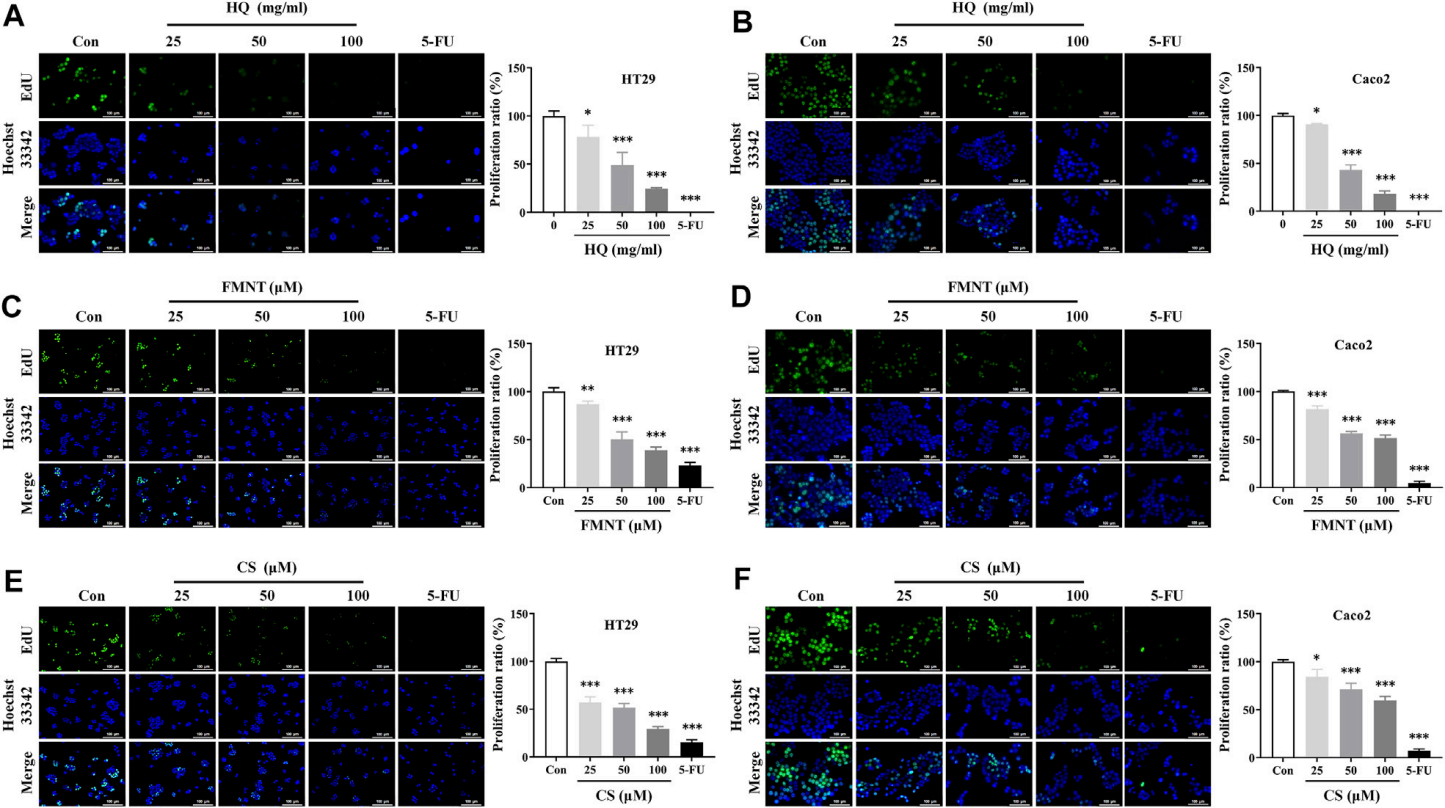
HQ, FMNT and CS significantly inhibited the proliferation of HT29 and Caco2 cells. The effects of HQ (25, 50 and 100 mg/mL, 48 h) **(A and B)**, FMNT (25, 50 and 100 μM, 48 h) **(C and D)** and CS (25, 50 and 100 μM, 48 h) **(E and F)** on the proliferation of HT29 and Caco2 cells, respectively. 5-FU was used as a positive control for inhibition of cell proliferation. The data represent the mean ± SD (*n* = 3). ^*^ *p* < 0.05, ^**^ *p* < 0.01 and ^***^ *p* < 0.001 compared with the control group.

EdU assay was used to further evaluate the effects of HQ, FMNT and CS on the proliferation of HT29 and Caco2 cells. As shown in Figures 6A, B, treatment with HQ at 25, 50, and 100 mg/mL for 48 h significantly decreased EdU positive cells in a dose-dependent manner over the control (*p* < 0.05 or *p* < 0.001), indicating that HQ could significantly suppressed the proliferation of HT29 and Caco2 cells. The number of EdU positive cells in the FMNT or CS treated groups were also significantly reduced in a dose-dependent manner (Figures 6C–F, *p* < 0.05, *p* < 0.01 or *p* < 0.001), indicating that both FMNT and CS significantly suppressed the proliferation of HT29 and Caco2 cells. 5-FU, as a positive control, markedly inhibited the proliferation of HT29 and Caco2 cells as expected (*p* < 0.001).

### 3.6 HQ, FMNT and CS significantly induced apoptosis of HT29 and Caco2 cells

**FIGURE 7:**
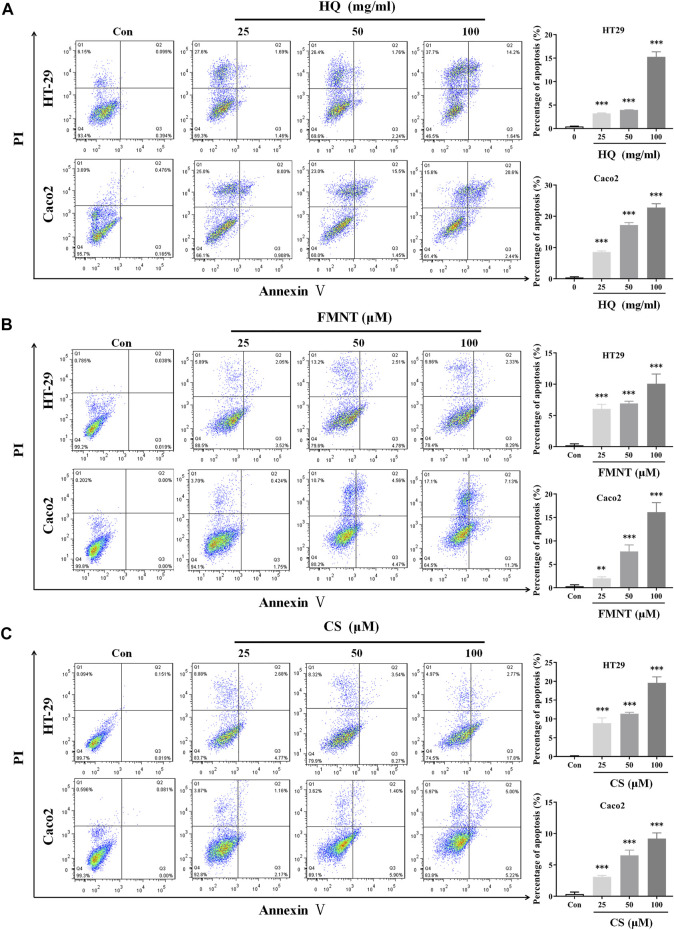
HQ, FMNT and CS significantly induced apoptosis of HT29 and Caco2 cells. The effects of HQ (25, 50 and 100 mg/mL, 48 h) **(A)**, FMNT (25, 50 and 100 μM, 48 h) **(B)** and CS (25, 50 and 100 μM, 48 h) **(C)** on the apoptosis of HT29 and Caco2 cells. The data represent the mean ± SD (*n* = 3). **p < 0.01 and ***p < 0.001 compared with the control group.

An Annexin V-FITC/PI staining assay was performed to evaluate whether HQ, FMNT and CS could induce apoptosis of HT29 and Caco2 cells. Treatment with HQ at 25, 50, and 100 mg/mL for 48 h significantly induced the apoptosis rate of HT29 and Caco2 cells in a dose-dependent manner (Figure 7A, *p* < 0.001). Apoptosis rate was also dose-dependently increased in HT29 and Caco2 cells exposed to FMNT (Figure 7B, *p* < 0.01 or *p* < 0.001) or CS (Figure 7C, *p* < 0.001) at 10, 20, and 40 μM for 48 h**.**

### 3.7 HQ, FMNT and CS significantly suppressed the migration of HT29 and Caco2 cells

**FIGURE 8:**
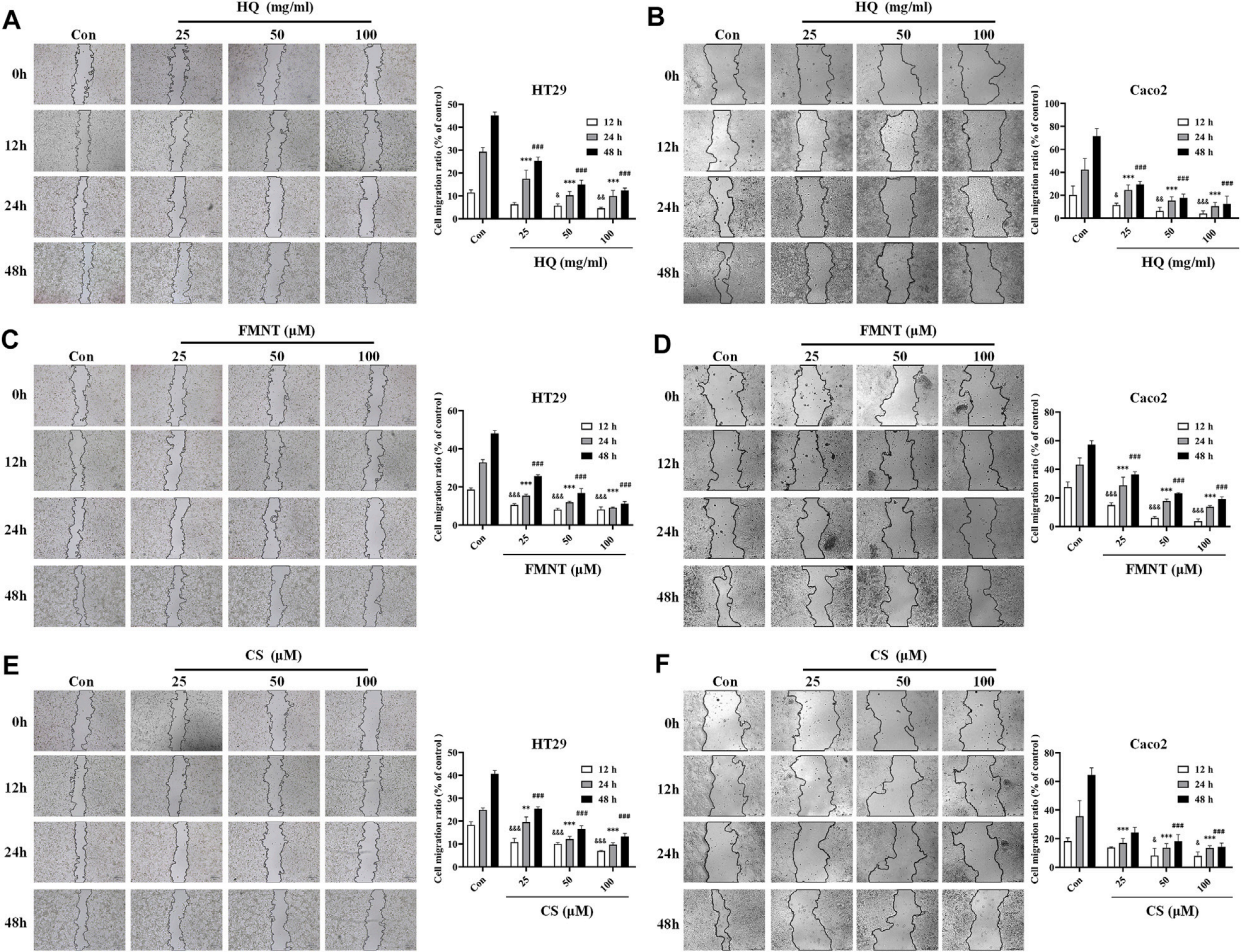
HQ, FMNT and CS significantly suppressed the migration of HT29 and Caco2 cells. The effects of HQ (25, 50 and 100 mg/mL) **(A and B)**, FMNT (25, 50 and 100 μM) **(C and D)** and CS (25, 50 and 100 μM) **(E and F)** on the migration of HT29 and Caco2 cells for 12, 24 and 48 h, respectively. The data represent the mean ± SD (*n* = 3). ^&^ *p* < 0.05, ^&&^ *p* < 0.01 and ^&&&^ *p* < 0.001 compared with the control group for 12 h incubation. ^***^ *p* < 0.001 compared with the control group for 24 h incubation. ^#^ *p* < 0.05, ^##^ *p* < 0.01 and ^###^ *p* < 0.001 compared with the control group for 48 h incubation.

Wound healing assay was used to evaluate the effects of HQ, FMNT and CS on the migration of HT29 and Caco2 cells. As shown in Figures 8A, B, treatment with HQ at 25, 50, and 100 mg/mL for 12–48 h significantly suppressed the migration ratio of HT29 and Caco2 cells dose-dependently over the control (*p* < 0.05, *p* < 0.01 or *p* < 0.001). Incubation of FMNT and CS at 10, 20, and 40 μM also exhibited significant inhibitory effects on the migration of HT29 and Caco2 cells (Figures 8C–F, *p* < 0.05, *p* < 0.01 or *p* < 0.001).

**FIGURE 9:**
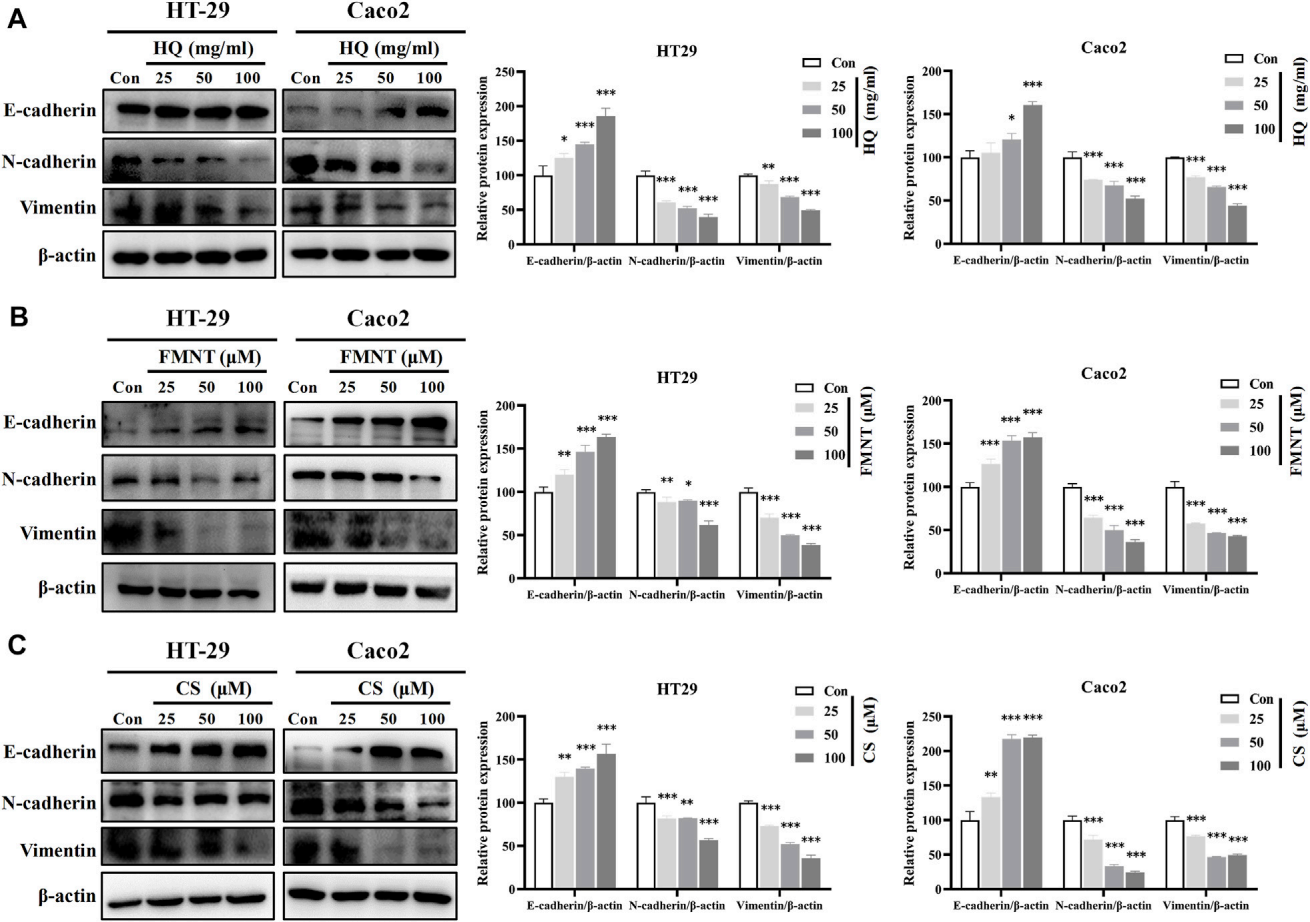
HQ, FMNT and CS significantly upregulated E-cadherin protein levels, and downregulated N-cadherin and Vimentin protein levels in HT29 and Caco2 cells. The protein levels of E-cadherin, N-cadherin, and Vimentin in HT29 and Caco2 cells were detected after treatment with HQ (25, 50 and 100 mg/mL, 48 h) **(A)**, FMNT (25, 50 and 100 μM, 48 h) **(B)** or CS (25, 50 and 100 μM, 48 h) **(C)**, respectively. The data represent the mean ± SD (*n* = 3). ^**^ *p* < 0.01 and ^***^ *p* < 0.001 compared with the control group.

**FIGURE 10:**
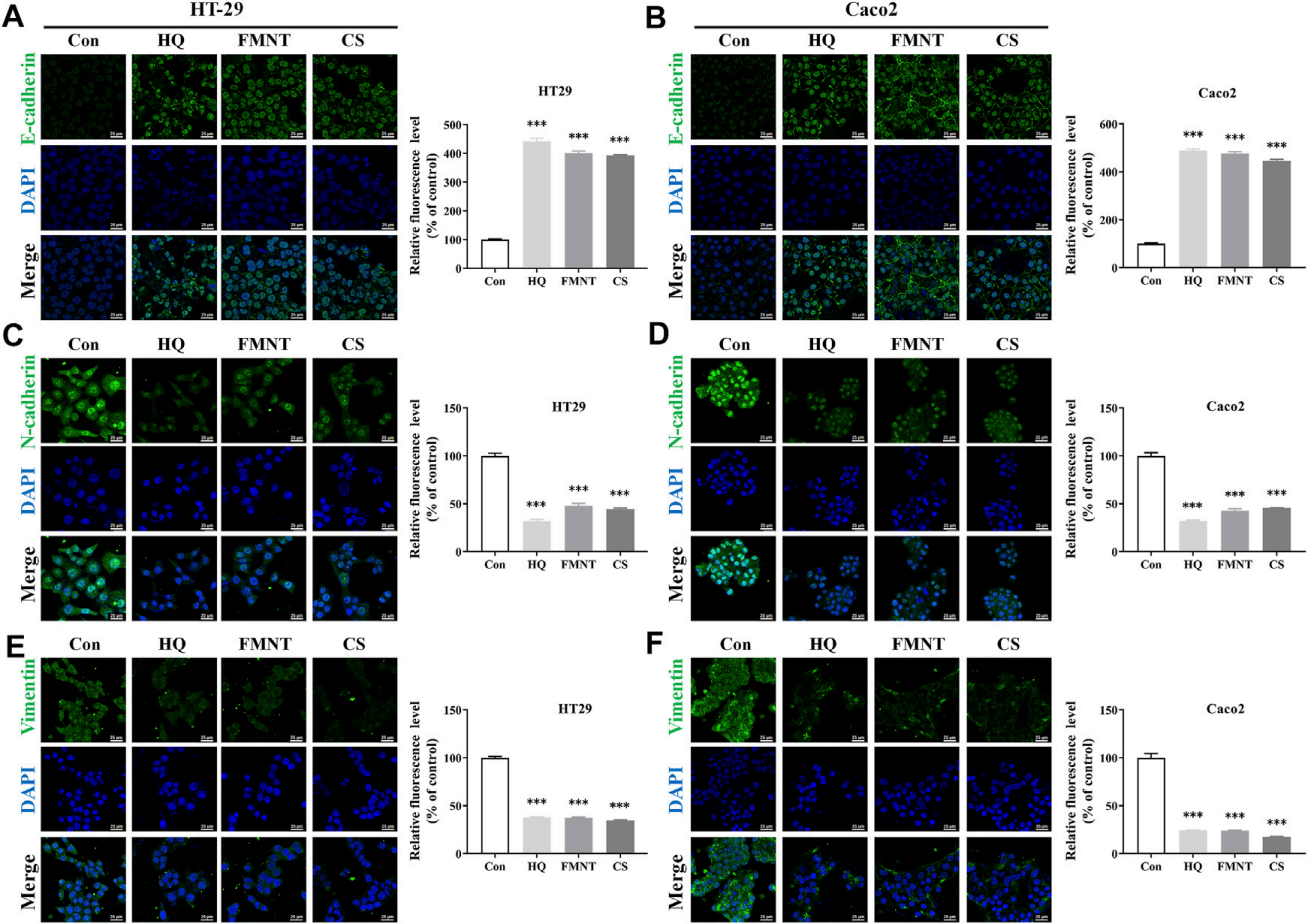
HQ, FMNT and CS significantly increased the fluorescence intensity of E-cadherin **(A, B)**, and decreased the fluorescence intensity of N-cadherin **(C, D)** and Vimentin **(E, F)** in HT29 and Caco2 cells. The fluorescence intensity of E-cadherin, N-cadherin, and Vimentin in HT29 and Caco2 cells were detected after treatment with HQ (100 mg/mL, 48 h), FMNT (100 μM, 48 h) or CS (100 μM, 48 h), respectively. The data represent the mean ± SD (*n* = 3). ^***^ *p* < 0.001 compared with the control group.

E-cadherin, N-cadherin, and Vimentin are critical epithelial–mesenchymal transformation (EMT)-related proteins, which that modulate cell migration and tumor invasiveness. E-cadherin is notably downregulated while N-cadherin and Vimentin are upregulated during EMT [30]. Hence, the intracellular protein levels of E-cadherin, N-cadherin, and Vimentin were further determined. As shown in Figure 9A, exposure to HQ at 25, 50, and 100 mg/mL for 48 h significantly upregulated the E-cadherin protein levels, while dose-dependently downregulated the protein levels of N-cadherin and Vimentin over the control (*p* < 0.05, *p* < 0.01, or *p* < 0.001). It was observed that incubation of FMNT and CS at 10, 20, and 40 μM for 48 h also strikingly increased the E-cadherin protein levels, while decreased the protein levels of N-cadherin and Vimentin in a dose-dependent manner (Figures 9B, C, *p* < 0.05, *p* < 0.01, or *p* < 0.001). Moreover, the results of immunofluorescence assay further revealed the expression of intracellular E-cadherin, N-cadherin, and Vimentin with the same treatment. Of note, the fluorescence intensity of E-cadherin was markedly enhanced, while the fluorescence intensity of N-cadherin and Vimentin was notably decreased in the cells exposed to HQ, FMNT and CS over the control cells (Figure 10).

### 3.8 HQ, FMNT and CS significantly inhibited the ERK1/2 and PI3K-Akt signaling pathways

**FIGURE 11:**
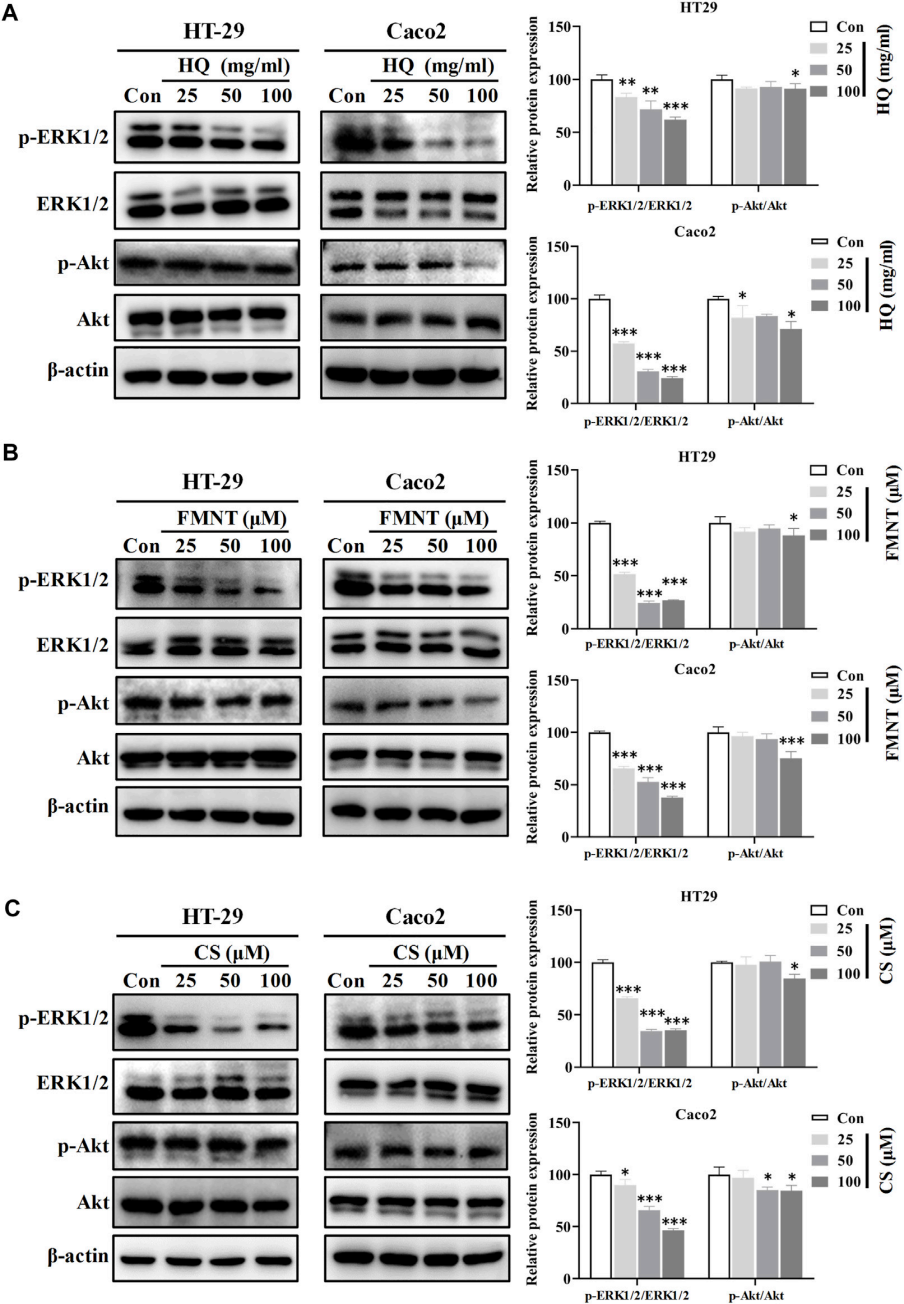
HQ, FMNT and CS significantly inhibited the ERK1/2 and PI3K-AKT signaling pathways. The protein levels of p-Erk1/2, ERK1/2, p-AKT and AKT in HT29 and Caco2 cells were detected after treatment with HQ (25, 50 and 100 mg/mL, 48 h) **(A)**, FMNT (25, 50 and 100 μM, 48 h) **(B)** or CS (25, 50 and 100 μM, 48 h) **(C)**, respectively. The data represent the mean ± SD (n = 3). ^*^ *p* < 0.05, ^**^ *p* < 0.01 and ^***^ *p* < 0.001 compared with the control group.

Western blot analysis was further performed to understand the roles of ERK1/2 and PI3K-Akt signaling in mediating the inhibitory effects of HQ, FMNT and CS against colon cancer. Compared with the control cells, exposure to HQ at 25, 50, and 100 mg/mL for 48 h strikingly downregulated the p-ERK1/2 expression in a dose-dependent manner (Figure 11A, *p* < 0.01, or *p* < 0.001), while did not notably alter the total ERK1/2 expression. Thus, p-ERK1/2/ERK1/2 ratio was significantly decreased in the cells exposed to HQ than that in the control cells (Figure 11A, *p* < 0.01, or *p* < 0.001). The treatment of HQ at high dose also slightly downregulated the p-AKT expression without marked change in total Akt expression. Similarly, Western blot analysis demonstrated a decreased p-ERK1/2 expression in the cells exposed to FMNT or CS (Figures 11B, C, *p* < 0.05 or *p* < 0.001). Thus, FMNT and CS treatment significantly decreased the p-ERK1/2/ERK1/2 ratio compared with the control cells (Figures 11B, C, *p* < 0.01, or *p* < 0.001). The same treatment of FMNT and CS at their high doses also slightly downregulated the p-Akt protein levels, while did not notably alter the total Akt expression (Figures 11B, C).

Person correlation analysis showed that the inactivation of HQ, FMNT and CS on ERK1/2 signaling pathway was closely related to their inhibitory effects against colon cancer. It was observed that HQ-induced decrease in ratio of p-ERK1/2/ERK1/2 was positively related to inhibition of cell viability, proliferation, and migration, while were negatively related to promotion of the cell apoptosis (Supplementary Figure S2, *p* < 0.001). Similarity, a strong positive correlation was found between the decrease in p-ERK1/2/ERK1/2 ratio and inhibition of cell viability, proliferation, and migration by FMNT and CS. Conversely, a significant negative correlation was observed between the decreased p-ERK1/2/ERK1/2 ratio and promotion of the cell apoptosis by FMNT and CS (Supplementary Figures S3, S4, *p* < 0.001).

### 3.9 FMNT and CS showed strong binding affinity for the core targets of EGFR/RAS/RAF/MEK/ERK pathway

**FIGURE 12:**
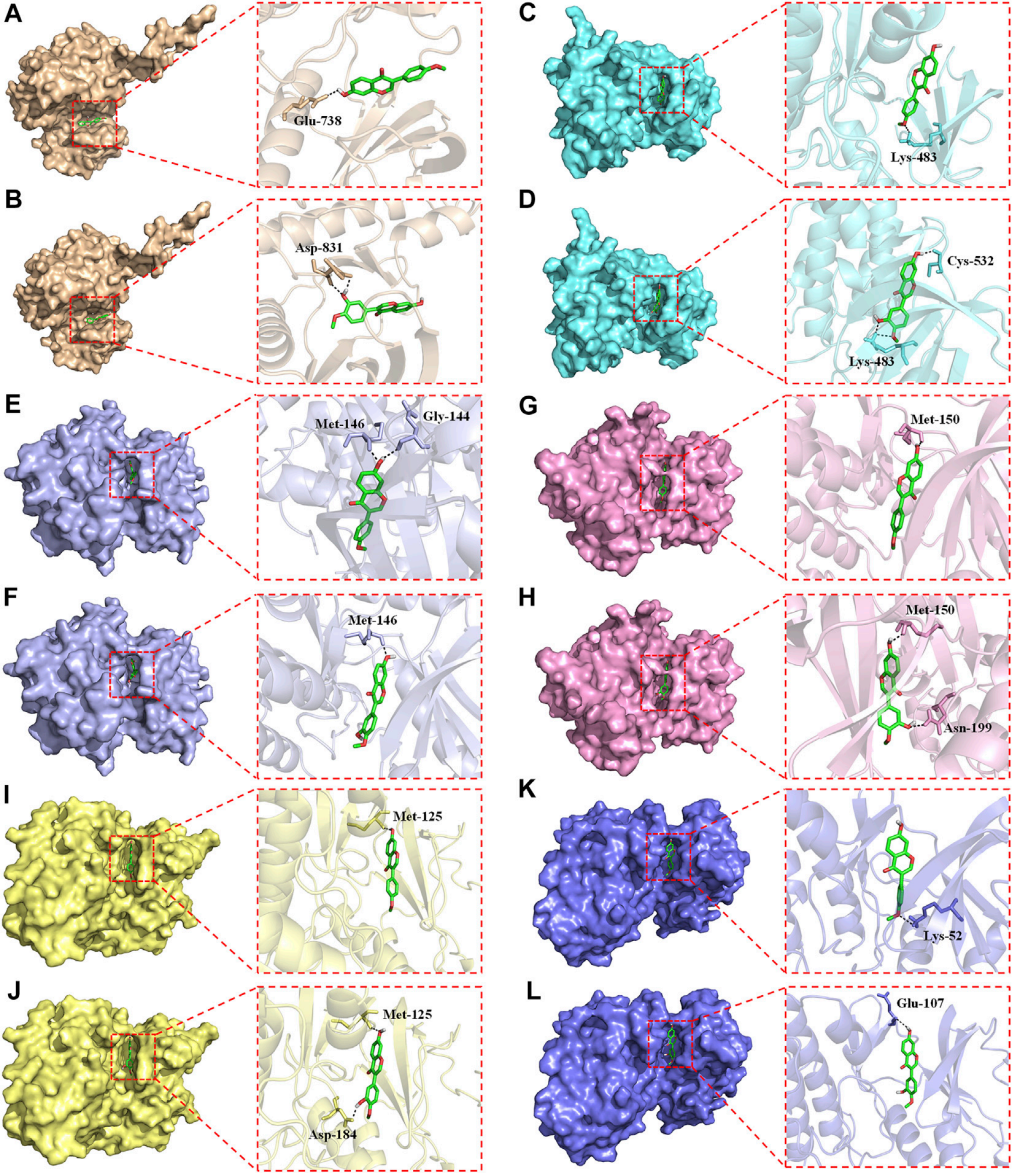
Pattern diagrams of molecular docking. FMNT-EGFR **(A)**. CS-EGFR **(B)**. FMNT-BRAF **(C)**. CS-BRAF **(D)**. FMNT-MEK1 **(E)**. CS-MEK1 **(F)**. FMNT-MEK2 **(G)**. CS-MEK2 **(H)**. FMNT-ERK1 **(I)**. CS-ERK1 **(J)**. FMNT-ERK2 **(K)**. CS-ERK2 **(L)**.

Overactivation of the RAS/RAF/MEK/ERK signaling pathway is associated with the carcinogenesis and progression of numerous cancers (Zhang et al., 2019; Zhu et al., 2020). Epidermal growth factor receptor (EGFR), a typical receptor tyrosine kinase, is activated by binding with ligands and takes part in cell differentiation, proliferation, migration, and apoptosis *via* activating the subsequent intracellular signaling pathways (Zhang et al., 2019). RAS/RAF/MEK/ERK is one of the most key EGFR downstream signaling pathways (Zhang et al., 2019). Thus, EGFR/RAS/RAF/MEK/ERK pathway is an essential target in clinical cancer therapy. Development of inhibitors of this pathway is considered as promising strategy for the prevention and treatment of numerous cancers (Supplementary Figure S5). Hence, molecular docking study was further conducted to explore the binding affinity of FMNT and CS with EGFR/RAS/RAF/MEK/ERK pathway. The pattern diagram of molecular docking was shown in Figure 12. Protein residues Glu738 and Asp831 in EGFR formed hydrogen bonds with FMNT and CS, respectively (Figures 12A, B). FMNT could bind to the protein residue Lys483, forming one single hydrogen bond (Figure 12C), while CS could bind to the protein residues Cys532 and Lys483 in BRAF through hydrogen bonds (Figure 12D). Protein residues Met146 and Gly144 in MEK1 formed hydrogen bonds with FMNT (Figure 12E), while only residue Met146 in MEK1 and CS bonded to each other through one single hydrogen bond (Figure 12F). FMNT could interact with the residue Met150 (Figure 12G), while CS could interact with the residues Met150 and Asn199 in MEK2 through hydrogen bonds (Figure 12H). Both FMNT and CS could interact with the residue Met125 in ERK1through hydrogen bonds (Figures 12I, J). CS could also interact with the residue Asp184 in ERK1 (Figure 12J). Protein residues Lys52 and Glu107 in ERK2 formed one single hydrogen bond with FMNT and CS, respectively (Figures 12K, L). The docking scores for FMNT and CS with the six crystal structures were showed in Table 1. The bond strength of hydrogen bonds represents the binding affinity between ligand compounds and receptor molecules, and when the docking score is lower, the binding affinity is stronger. An affinity <−7 indicates strong binding activity (Trott and Olson, 2010). Thus, the results revealed that FMNT and CS have a strong binding affinity for the key targets of EGFR/RAS/RAF/MEK/ERK pathway, and the binding affinity of CS with the targets was stronger than that of FMNT to the same targets.

## 4 Discussion

Worldwide, colon cancer is the third most frequent malignant disease with high morbidity, recurrence and mortality (Ferlay et al., 2019; Kong et al., 2020). Despite advances in surgery, chemotherapy, radiotherapy and immunotherapy, the prognosis in colon cancer patients remains poor and the recurrence rate is still high (Kong et al., 2020). What is more, long-term use of these therapies will easily produce undesirable side effects and toxicologically unsafe consequences (Kong et al., 2020). CHMs have played an indispensable role in healthcare throughout Chinese history, and have also been increasingly used worldwide for prevention and treatment of various diseases. Substantial evidence exists that CHMs are effective and safe as alternative remedies for patients with colon cancer. However, the applications of many CHMs in the clinical treatment of colon cancer is also greatly restricted because of their multiple ingredients and different mechanisms of action (Yan et al., 2017; Guo et al., 2019). Hence, it is urgent and necessary to fully elucidate the effects and molecular mechanisms to promote the applications of CHMs for healthcare all over the world.

In recent years, network pharmacology has been increasingly applied to expand our knowledge pertaining to the multiple targets and mechanisms of CHMs in the clinical treatment of various diseases, including numerous cancers (Li and Zhang, 2013; Hao da and Xiao, 2014). Network pharmacology, a new discipline which integrates network biology and polypharmacology approaches, is able to reveal drug properties and interactions with multiple protein targets. Hence, network pharmacology can provide beneficial information for studying the modulation of various complex biological, physiological, and pathological systems (Berger and Iyengar, 2009; Li and Zhang, 2013; Hao da and Xiao, 2014). Currently, network pharmacology has been frequently used as a powerful tool to elucidate the complex and holistic mechanisms of CHMs (Li and Zhang, 2013; Hao da and Xiao, 2014). Therefore, we first screened the potential targets of HQ and its bioactive ingredients FMNT and CS against colon cancer, as well as investigated the mechanism using network pharmacology analysis. 147 potential targets of FMNT and CS in the suppression of colon cancer were obtained (Figure 2). 19 core targets were further screened and analyzed. It was apparent that the 19 key targets, included AKT1, TP53, MAPK3, EGFR, HSP90AA1, ESR1, MYC, CASP3, TNF, JUN, PTEN, MAPK1, FOS, RELA, FN1, PTGS2, HSP90AB1, MAPK8 and PPARG, were closely involved in cell proliferation, differentiation, migration, and apoptosis. Next, GO enrichment analysis proposed that FMNT and CS could exhibit inhibitory activity against colon cancer by simultaneously activating various BPs, CCs, and MFs (Figure 3). KEGG pathway enrichment further indicated that multiple pathways including pathways in cancer, MAPK and PI3K-Akt signaling pathways could be associated with the inhibitory effects of FMNT and CS against colon cancer (Figure 4). Accordingly, we next conducted experimental validation to elucidate the mechanism of HQ, FMNT and CS against colon cancer.

Previous studies showed that HQ and its isoflavonoids have pharmacological effects against colon cancer. HQ can not only inhibit the proliferation, cause cell cycle arrest and promote apoptosis of colon cancer cells, but also inhibit the migration of colon cancer cells (Tseng et al., 2016; Tan et al., 2019). HQ could effectively inhibit the tumors of human colon cancer cells grafted into nude mice without obvious unsafe consequences (Tseng et al., 2016), which demonstrated that HQ might be a potential chemopreventive drug for therapy colon cancer. The study of HQ in controlling angiogenesis revealed that HQ exerted anti-tumor activity in colon cancer cells *via* modulation of mTOR and COX-2 signaling, which together reduced VEGF expression to suppress angiogenesis in human CRC cell and tumor xenograft (Auyeung et al., 2016). FMNT was also proven to downregulate the expression of key pro-angiogenic factors such as VEGF and matrix metalloproteinases in colon cancer cells, and to inhibit angiogenesis and tumor cell invasion, thereby supporting its use in advanced and metastatic colon therapies (Auyeung et al., 2012). In addition to FMNT, CS could notably inhibited proliferation and induced apoptosis of colon cancer cells through regulating multiple signaling, including ERβ/MiR-95, ERβ/miR-17, IGF-1R and PI3K-Akt signaling pathways (Chen et al., 2015b; Zhao et al., 2016), which indicates that CS serves as an attractive chemotherapeutic agent against colon cancer. The results from this study demonstrated that HQ, FMNT and CS could effectively inhibit the viability and proliferation, as well as promote apoptosis of HT29 and Caco2 cells (Figures 5–7), suggesting that FMNT and CS could be the beneficial ingredients responsible for the therapeutic efficacy of HQ in the suppression of colon cancer.

Growing evidence suggests that invasion and metastasis are the fundamental reason of colon cancer treatment failure and the leading cause of death in colon cancer patients (Wen et al., 2022). Suppression of invasion and metastasis is of great therapeutic interest in counteracting chemoresistance in patients with colon cancer. The proliferation of tumor cells was the basis of tumor invasion and metastasis (Yang et al., 2019). This current study showed that HQ, FMNT and CS could dose-dependently inhibit the proliferation of colon cancer cells (Figure 6). Thus, the wound healing assay was conducted to further confirm the impact of HQ, FMNT and CS on the metastasis of colon cancer cells. It was found that HQ, FMNT and CS could significantly suppress the migration of colon cancer cells in dose- and time-dependent manners (Figure 8), indicating that HQ, FMNT and CS could inhibit the metastasis of colon cancer cells. It is well-known that E-cadherin, N-cadherin, and Vimentin are critical EMT-related proteins, which play pivotal roles in modulation of cell migration and tumor invasiveness (Hazan et al., 2000). E-cadherin, a calcium-dependent cell-cell adhesion molecule, takes part in epithelial cell behavior, tissue formation, and cancer suppression (van Roy and Berx, 2008). N-cadherin, which serves as an indicator of ongoing EMT, is closely associated with the development of diverse cancers (Loh et al., 2019). Vimentin, as a typical marker for EMT, is overexpressed in various epithelial cancers and correlates well with accelerated tumor growth, invasion, and poor prognosis (Satelli and Li, 2011). During EMT, N-cadherin and Vimentin are upregulated while E-cadherin is downregulated. Hence, the expression of intracellular E-cadherin, N-cadherin, and Vimentin were further determined. It was found that HQ, FMNT and CS could strikingly upregulate the E-cadherin protein levels, while simultaneously downregulated the protein levels of N-cadherin and Vimentin in a dose-dependent manner (Figure 9). In addition, immunofluorescence assay revealed that the fluorescence intensity of E-cadherin was markedly increased, while the fluorescence intensity of N-cadherin and Vimentin was notably decreased (Figure 10). These data further indicate that HQ, FMNT and CS could effectively suppress colon cancer by inhibiting the metastasis of colon cancer cells.

In this study, the network pharmacological study found that the MAPK and PI3K-Akt signaling pathways were two important pathways for HQ in treating colon cancer. MAPK signaling pathway has a crucial role in cell proliferation, differentiation, migration, and apoptosis, including phosphorylation of ERK1/2, P38, and JNK (Haigis et al., 2008; Ahearn et al., 2011). ERK1/2, one of the best-characterized members of MAPK family, mediates a wide range of cellular responses like gene expression, metabolism, motility, cell survival and death (Wang et al., 2022). Substantial evidence exists that the phosphorylation of ERK1/2 could increase cell proliferation and cause an anti-apoptotic effect, thereby promoting the progression of tumor invasion and metastasis (Lebedev et al., 2022; Wang et al., 2022). Conversely, suppression of ERK1/2 could effectively block many other extracellular signals that promote cell growth and movement, ultimately suppressing tumor invasion and metastasis (Lebedev et al., 2022; Wang et al., 2022). Hence, identification of small molecules that can inhibit ERK1/2 activity may be a useful pharmacological strategy for the clinical treatment of numerous cancers (Lebedev et al., 2022; Wang et al., 2022). In addition to ERK1/2, the PI3K-Akt signaling pathway also takes part in a variety of cellular activities, including cell growth, metabolism, motility, proliferation, survival, and apoptosis (Porta et al., 2014). Aberrant activation of the PI3K-Akt pathway promotes the growth and proliferation of tumor cells in various types of human cancers (Huang and Hung, 2009; Porta et al., 2014). Knockout or suppression of PI3K-Akt pathway has been an attractive strategy to effectively inhibit tumor growth and invasiveness (Cheng et al., 2005). The data of this study displayed that HQ, FMNT and CS significantly decreased the intracellular p-ERK1/2 expression in a dose-dependent manner, while did not notably alter the total ERK1/2 expression (Figure 11). The same treatment of HQ, FMNT and CS at their high doses also slightly downregulated the intracellular p-AKT expression without marked alteration in total Akt expression (Figure 11). Person correlation analysis revealed that HQ, FMNT and CS-induced decrease in p-ERK1/2/ERK1/2 ratio was positively related to their inhibition of cell viability, proliferation, and migration, while were negatively related to their promotion of the cell apoptosis (Supplementary Figures S2-S4). These results suggested that HQ, FMNT and CS could manifest remarkable inhibitory activity against colon cancer mainly *via* reducing the phosphorylation of ERK1/2 to inhibit the ERK1/2 pathway. This suppression of colon cancer by HQ, FMNT and CS was also partially mediated *via* inactivation of the PI3K-Akt signaling pathway. Finally, to validate the potential mechanism of HQ, FMNT and CS against colon cancer, molecular docking was further performed to explore the binding affinity of FMNT and CS with upstream molecules of ERK1/2 pathway. As protein-serine/threonine kinases, ERK1 and ERK2 participate in the RAS/ARF/MEK/ERK signal transduction cascade that play a crucial role in the modulation of a large variety of cellular activities including cell adhesion, metabolism, cell survival, cell migration, cell cycle progression, proliferation, differentiation and transcription (Roskoski, 2012). Overactivation of the RAS/ARF/MEK/ERK signaling is correlated with the carcinogenesis and progression of numerous cancers (Zhang et al., 2019; Zhu et al., 2020). Epidermal growth factor receptor (EGFR), a typical receptor tyrosine kinase, is activated by binding with ligands and takes part in cell survival, proliferation, migration, differentiation, and apoptosis *via* activating the subsequent intracellular signaling pathways (Zhang et al., 2019). RAS/ARF/MEK/ERK is one of the most key EGFR downstream signaling pathways (Zhang et al., 2019). Development of inhibitors that target EGFR/RAS/ARF/MEK/ERK pathway is considered an effective and promising strategy for the clinical treatment of numerous cancers (Supplementary Figures S5). The pattern diagram of molecular docking showed that different amino acid residues in the crystal structures of EGFR, RAS, ARF, MEK or ERK formed hydrogen bonds with FMNT and CS, respectively (Figure 12). The bond strength of hydrogen bonds represents the binding affinity between ligand compounds and receptor molecules, and when the docking score is lower, the binding affinity is stronger. An affinity <−7 suggests strong binding activity (Trott and Olson, 2010). The results of molecular docking study indicate that FMNT and CS have a strong binding affinity for the key targets of EGFR/RAS/ARF/MEK/ERK pathway, and the binding affinity of CS with the targets was stronger than that of FMNT to the same targets (Table 1). These finding indicated that the mechanism of the HQ, FMNT and CS against colon cancer might be due to suppression of the ERK1/2 pathway through potent inhibition of its upstream molecules. Further studies are needed to determine the exact mechanism(s) of the regulation of HQ, FMNT and CS on the ERK1/2 signaling pathway. Further animal experimental research on the inhibitory effects of HQ, FMNT and CS against colon cancer needs to be continued.

## 5 Conclusion

This study investigated the effects and mechanism of HQ and its bioactive ingredients FMNT and CS against colon cancer using network pharmacology analysis coupled with experimental validation and molecular docking. The findings suggested that the HQ exerted good therapeutic effects against colon cancer by mainly inhibiting the ERK1/2 signaling pathway. FMNT and CS were two bioactive ingredients responsible for the inhibitory effects of HQ against colon cancer. The current study expands our knowledge pertaining to the effects and mechanism of HQ against colon cancer, and suggests that FMNT and CS will hopefully serve as prospective compounds for colon cancer treatment.

## Data Availability

The original contributions presented in the study are included in the article/Supplementary Material, further inquiries can be directed to the corresponding authors.

## Abbreviations

CHMs, Chinese herbal medicines; HQ, *Astragalus membranaceus* (Fisch.) Bunge (Huang Qi); FMNT, Formononetin; CS, Calycosin; PPI, Protein-protein interaction; GO, Gene Ontology; KEGG, Kyoto Encyclopedia of Genes and Genomes; BP, Biological process; CC, cellular component; MF, Molecular function; 5-FU, 5-Fluorouracil; IC_50_, 50% inhibitory concentration; DC, Degree centrality; EMT, Epithelial–mesenchymal transformation; EGFR, Epidermal growth factor receptor; MAPK, Mitogen-activated protein kinase; mTOR, Mammalian target of rapamycin; COX-2, Cyclooxygenase-2; VEGF, vascular endothelial growth factor.
