# Supplementary material for: Uncovering the effects and molecular mechanism of Astragalus membranaceus (Fisch.) Bunge and its bioactive ingredients formononetin and calycosin against colon cancer: An integrated approach based on network pharmacology analysis coupled with experimental validation and molecular docking
Source: Front Pharmacol. 2023 Jan 23;14:1111912. doi: 10.3389/fphar.2023.1111912 (PMC9899812; doi:10.3389/fphar.2023.1111912)
Supplement: Supplementary file 1 [file DataSheet1.docx]

**Supplementary Materials and Methods**

**UHPLC-MS/MS analysis**

UHPLC-MS/MS analysis was performed on Agilent 1290 infinity LC system using a SB - C18 column (1.8 µm, 3.0 × 100 mm, Agilent, USA). A gradient consisting of solvent A (0.1% HCOOH) and solvent B (acetonitrile) was applied at a flow rate of 0.3 mL/min as follows: 0 - 1.5 min 30% - 90% B; 1.5 - 3.5 min 90% - 90% B; 3.5 - 4 min 90% - 100% B; 4 -5 min 100% - 100% B; 5 - 5.5 min 100% - 30% B. The injection volume was 5 µL and the electrospray interface was ESI source. MS parameters were set as follows: gas temperature, 380 ^°^C; nebulizer pressure, 500 V; drying gas (nitrogen), 5 liters/min; capillary voltage, 3500 V; fragmentor voltage, 200 V. The ionization of all compounds was performed using an electrospray interface operating in the positive mode in the multiple monitoring mode (MRM). The monitor ion pairs and collision voltages (CE) as shown in Supplementary Table 1. Quantification was performed based on peak areas using erlotinib (IS) of standard prepared in methanol.

**Supplementary Table 1. Parameters for multi-reaction monitoring of compounds**

| Compounds | Transition | Fragmentor | CE (eV) | Polarity |
| --- | --- | --- | --- | --- |
| Calycosin | 284.9→268.9 | 110 | 27 | Positive |
| Formononetin | 269.1→197 | 135 | 45 | Positive |
| Erlotinib (IS) | 394.2→278.1 | 135 | 32 | Positive |

**Supplementary Figure legends**

**Supplementary Figure 1.** Schematic drawing to illustrate the MAPK (A) and PI3K-AKT (B) signaling pathways involving FMNT and CS against colon cancer. The key targets involved in this process are colored in green.

**Supplementary Figure 2. Pairwise correlation analysis.** Heat map of the cell viability, proliferation, migration, apoptosis, p-ERK1/2/ERK1/2 ratio, expression of E-cadherin, N-cadherin and Vimentin in HT29 (A) and Caco2 (B) cells after treatment with HQ (25, 50 and 100 mg/L, 48 h). Pairwise correlation between p-ERK1/2/ERK1/2 ratio and cell viability, proliferation, apoptosis, migration, expression of E-cadherin, N-cadherin and Vimentin, respectively, in HT29 (C) and Caco2 (D) cells.

**Supplementary Figure 3. Pairwise correlation analysis.** Heat map of the cell viability, proliferation, migration, apoptosis, p-ERK1/2/ERK1/2 ratio, expression of E-cadherin, N-cadherin and Vimentin in HT29 (A) and Caco2 (B) cells after treatment with FMNT (25, 50 and 100 μM, 48 h). Pairwise correlation between p-ERK1/2/ERK1/2 ratio and cell viability, proliferation, apoptosis, migration, expression of E-cadherin, N-cadherin and Vimentin, respectively, in HT29 (C) and Caco2 (D) cells.

**Supplementary Figure 4. Pairwise correlation analysis.** Heat map of the cell viability, proliferation, migration, apoptosis, p-ERK1/2/ERK1/2 ratio, expression of E-cadherin, N-cadherin and Vimentin in HT29 (A) and Caco2 (B) cells after treatment with CS (25, 50 and 100 μM, 48 h). Pairwise correlation between p-ERK1/2/ERK1/2 ratio and cell viability, proliferation, apoptosis, migration, expression of E-cadherin, N-cadherin and Vimentin, respectively, in HT29 (C) and Caco2 (D) cells.

**Supplementary Figure 5.** Schematic drawing of inhibitors of EGFR/RAS/RAF/MEK/ERK signaling pathway. EGFR/RAS/RAF/MEK/ERK pathway is an essential target in clinical cancer therapy. Development of inhibitors of this pathway is considered as a promising strategy for the prevention and treatment of numerous cancers.

**Supplementary Figure 1**

**
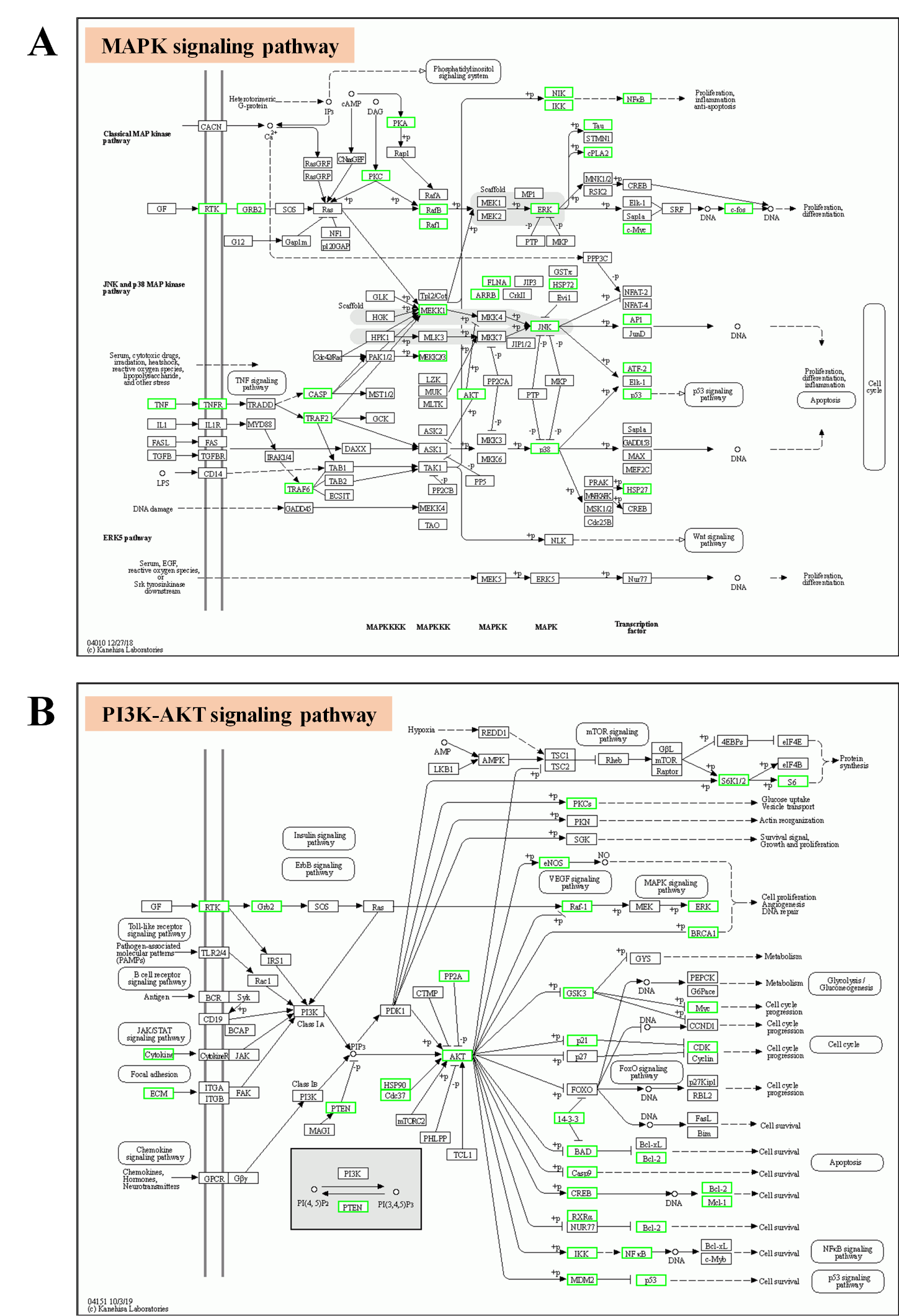
**

**Supplementary Figure 2**

**
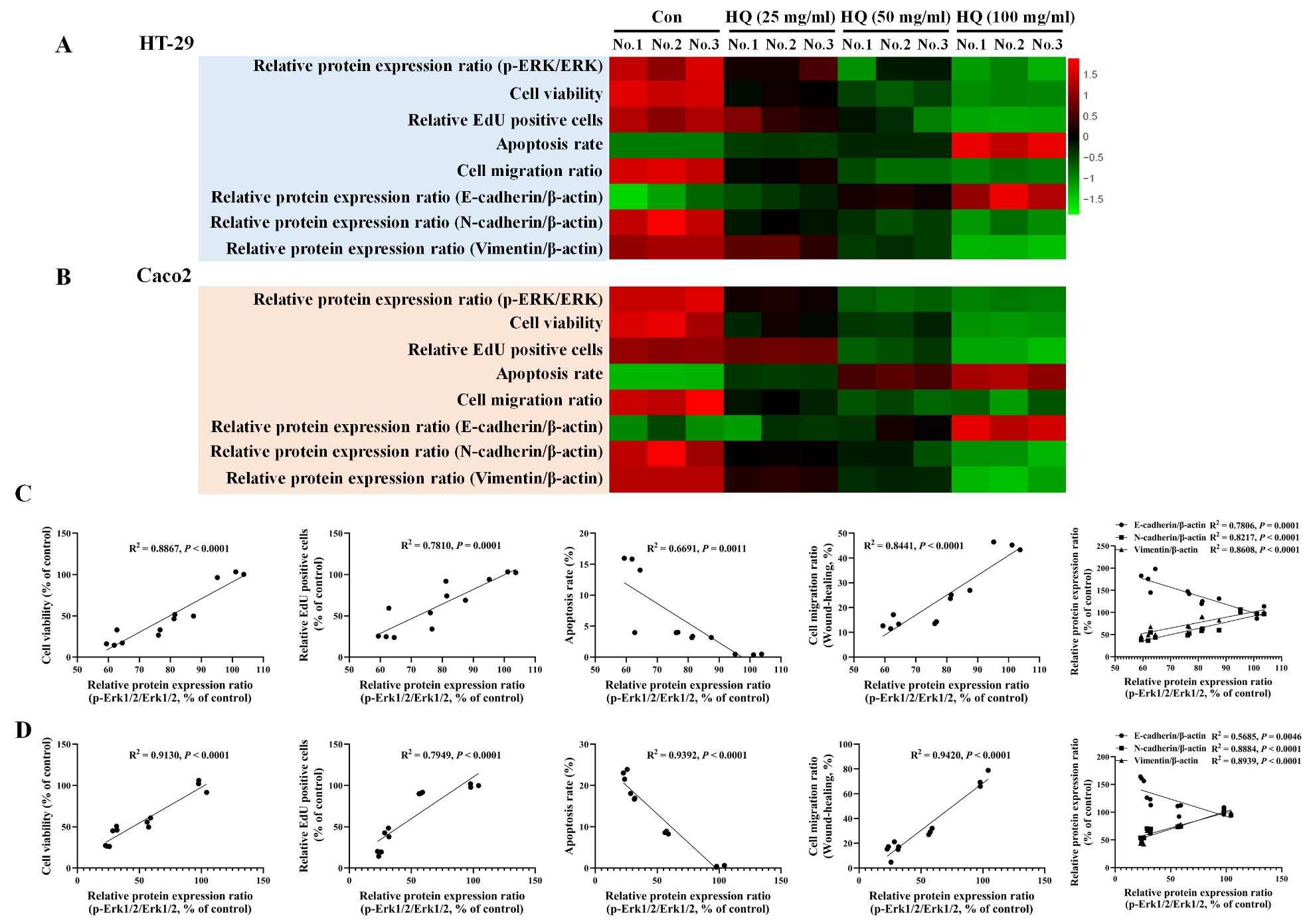
**

**Supplementary Figure 3**

**
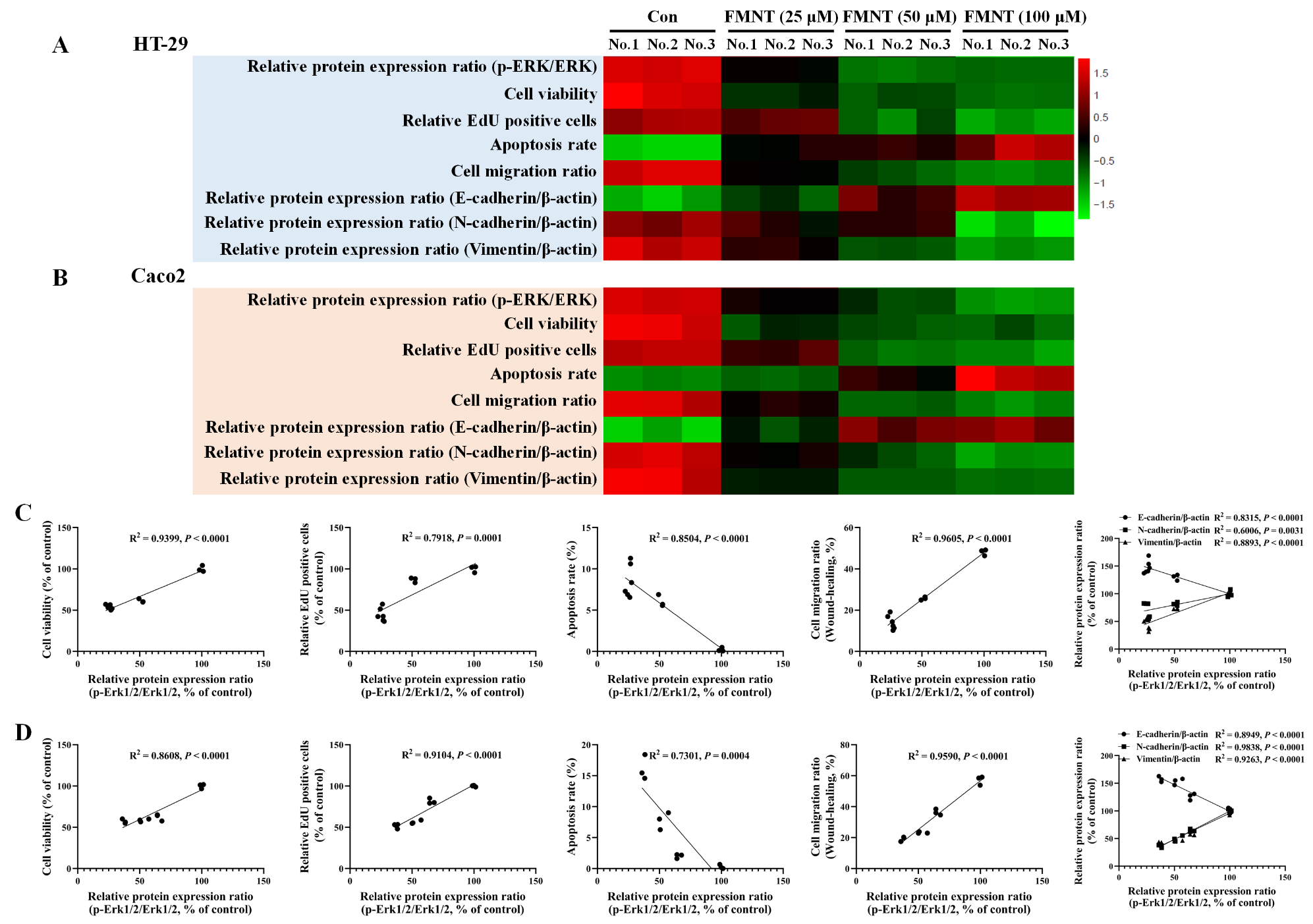
**

**Supplementary Figure 4**

**
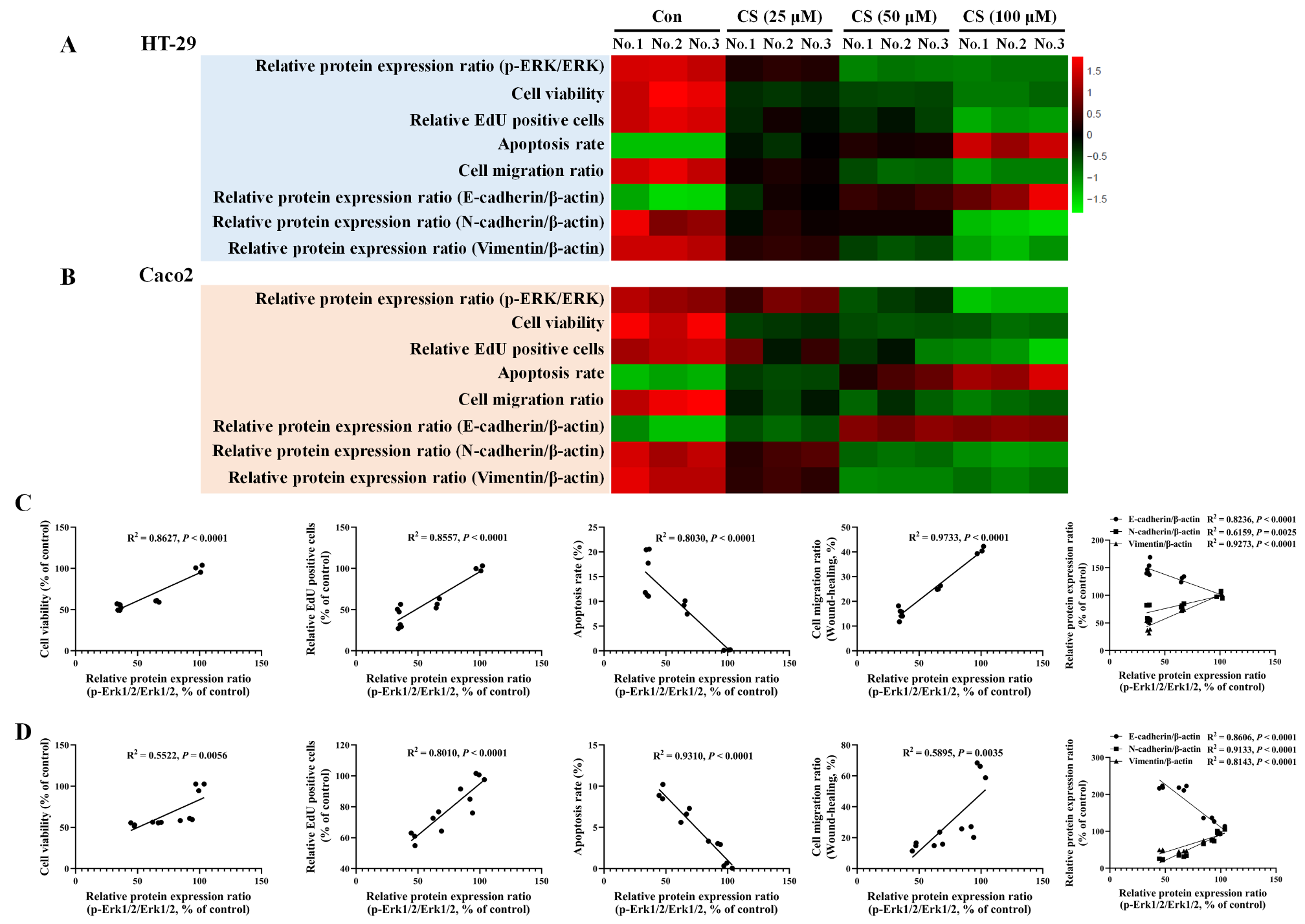
**

**Supplementary Figure 5**

**
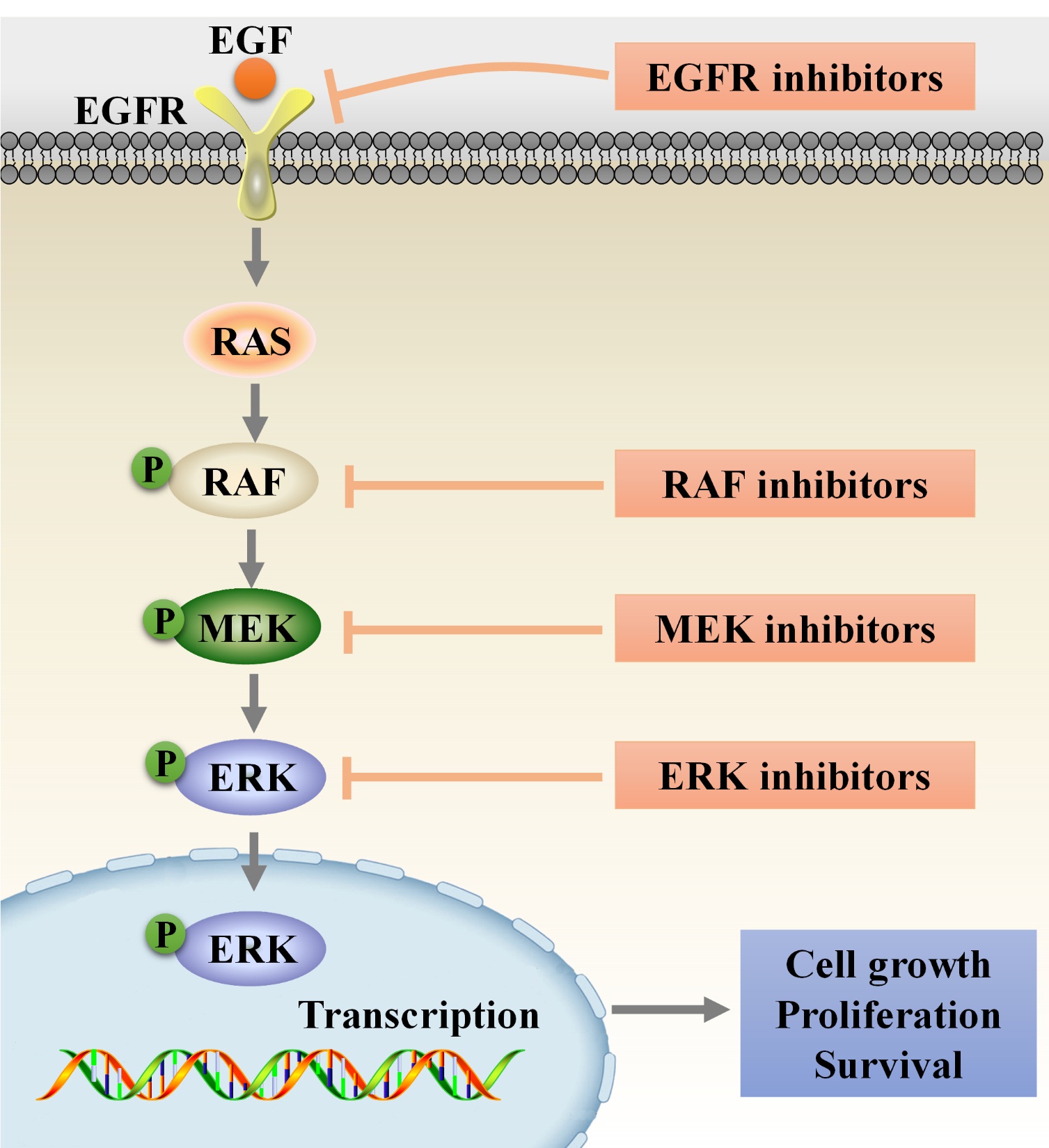
**
